# An NLRP3 inflammasome-triggered cytokine storm contributes to Streptococcal toxic shock-like syndrome (STSLS)

**DOI:** 10.1371/journal.ppat.1007795

**Published:** 2019-06-06

**Authors:** Lan Lin, Lei Xu, Weihua Lv, Li Han, Yaozu Xiang, Lei Fu, Meilin Jin, Rui Zhou, Huanchun Chen, Anding Zhang

**Affiliations:** 1 State Key Laboratory of Agricultural Microbiology, College of Veterinary Medicine, Huazhong Agricultural University, Wuhan, Hubei, China; 2 Key Laboratory of Preventive Veterinary Medicine in Hubei Province, The Cooperative Innovation Center for Sustainable Pig Production, Wuhan, Hubei, China; 3 Shanghai East Hospital, School of Life Sciences and Technology, Tongji University, Shanghai, China; 4 Key Laboratory of Development of Veterinary Diagnostic Products, Ministry of Agriculture of the People’s Republic of China, Wuhan, Hubei, China; 5 International Research Center for Animal Disease, Ministry of Science and Technology of the People’s Republic of China, Wuhan, Hubei, China; University of Toronto, CANADA

## Abstract

Infection with the *Streptococcus suis* (*S*. *suis*) epidemic strain can cause Streptococcal toxic shock-like syndrome (STSLS), which is characterized by a cytokine storm, dysfunction of multiple organs and a high incidence of mortality despite adequate treatment. Despite some progress concerning the contribution of the inflammatory response to STSLS, the precise mechanism underlying STSLS development remains elusive. Here, we use a murine model to demonstrate that caspase-1 activity is critical for STSLS development. Furthermore, we show that inflammasome activation by *S*. *suis* is mainly dependent on NLRP3 but not on NLRP1, AIM2 or NLRC4. The important role of NLRP3 activation in STSLS is further confirmed *in vivo* with the NLRP3 inhibitor MCC950 and *nlrp3*-knockout mice. By comparison of WT strain with isogenic strains with mutation of various virulence genes for inflammasome activation, Suilysin is essential for inflammasome activation, which is dependent on the membrane perforation activity to cause cytosolic K^+^ efflux. Moreover, the mutant strain msly (P353L) expressing mutagenic SLY without hemolytic activity was unable to activate the inflammasome and does not cause STSLS. In summary, we demonstrate that the high membrane perforation activity of the epidemic strain induces a high level of NLRP3 inflammasome activation, which is essential for the development of the cytokine storm and multi-organ dysfunction in STSLS and suggests NLRP3 inflammasome as an attractive target for the treatment of STSLS.

## Author summary

The two large-scale human *Streptococcus suis* epidemics have caused unusual development of Streptococcal Toxic-Shock-like Syndrome (STSLS) and high incidence of mortality despite adequate treatments. However, how the epidemic strain causes STSLS remained to be elucidated. Because an excessive high level of inflammasome-regulated cytokine was detected in the blood of STSLS patients, we used a murine model to identify the role of inflammasome activation on the development of STSLS. We found that NLRP3 activation contributed to STSLS with the pharmacological inhibition and NLRP3^-/-^ mice. We identified a novel mechanism of STSLS in that increased suilysin expression in *S*. *suis* highly virulent strain could induce high level of cytosolic K^+^ efflux, an essential event for NLRP3 inflammasome activation, and then further cause a cytokine storm, dysfunction of multiple organs and a high incidence of mortality, the characters of STSLS. Therefore, our study provides insights for STSLS development and highlights NLRP3 inflammasome as an attractive target for the treatment of STSLS.

## Introduction

*Streptococcus suis* (*S*. *suis*) is a major swine pathogen that is responsible for severe economic losses in the porcine industry and represents a significant threat to human health [1–4]. To date, more than 1600 human *S*. *suis* infections have been reported worldwide [4, 5], and the infection has been identified as the leading and second-leading cause of adult meningitis in Vietnam and Thailand [2]. *S*. *suis* infection mainly induces meningitis, sepsis, arthritis, endocarditis, and endophthalmitis, and the pooled case-fatality rate is 12.8% [5]. However, two large-scale human *S*. *suis* epidemics in China (the first was 25 cases with 14 deaths in Jiangsu in 1998, and the second was 204 cases with 38 deaths in Sichuan in 2005) raised serious concerns for global public health and challenged the conventional perception that *S*. *suis* infections are sporadic in humans [2, 6, 7]. This infection causes unusual development of Streptococcal toxic-shock-like syndrome (STSLS), including the hallmarks of acute high fever, blood spots, hypotension, shock, and dysfunction of multiple organs, as well as acute death (mortality is more than 80% despite adequate treatment) [7, 8].

At present, how the epidemic strain causes STSLS and leads to high mortality remains unclear. A retrospective clinical investigation showed high tumor necrosis factor-alpha (TNF-α), interleukin (IL)-1β, IL-6, IL-8, IL-12, and interferon-γ (IFN-γ) levels in the blood of patients with STSLS [6]. Subsequent studies further confirmed that the induction of an inflammatory cytokine storm was essential for STSLS [9, 10], which was further supported by the finding that inhibition of the excessive inflammatory response with anti-inflammatory drugs improved survival against STSLS [11]. Together, these data highlight the great potential that comprehensive understanding of the molecular mechanisms by which *S*. *suis* induces a high level of inflammatory responses may contribute to identify new therapeutic targets for *S*. *suis*-caused conditions, including STSLS [11, 12].

IL-1β secretion is tightly controlled by the assembly of a multiprotein complex called the inflammasome [13, 14]. To date, a few types of inflammasomes (NLRP1, NLRP3, NLRC4, AIM2, etc.) have been described, and the NLRP3 inflammasome has been under intense investigation given its link with a vast number of diseases [13, 15, 16]. Upon activation, NLRP3 is recruited to the dispersed trans-Golgi network to form multiple puncta that induces ASC polymerization and makes pro-caspase-1 (pro-casp1) into an active protease [17]. In turn, caspase-1 (casp1) mediates the processing of several targets, including pro-IL-1β and pro-IL-18, into their biologically active forms and induces their secretion by triggering pyroptosis through cleaved gasdermin D (GSDMD) [18–21]. IL-1β and IL-18 secretion may further induce IL-6, IL-8, IL-17, and IFN-γ expression, thereby resulting in inflammatory conditions such as fever and septic shock [22]. Owing to the high levels of blood IL-1β and its inflammatory mediators in patients with STSLS [6], we hypothesized that the inflammasome could contribute to STSLS. Here, we demonstrated for the first time that a high level of inflammasome activation was essential for induction of the cytokine storm and the dysfunction of multiple organs—the hallmarks of STSLS.

## Results

### A potential critical role of inflammasome activation in STSLS

**Fig 1:**
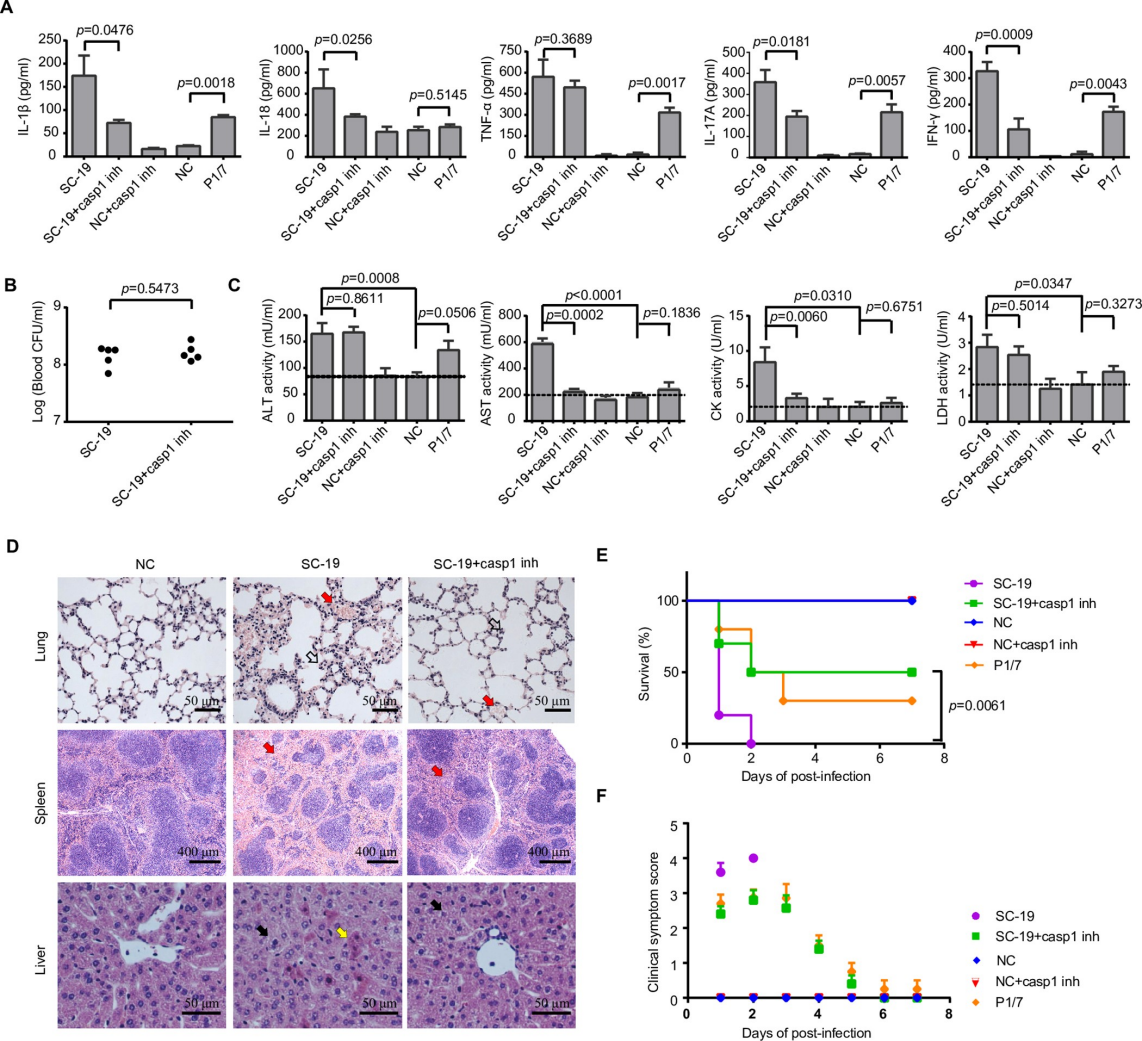
Inflammasome activation contributed to STSLS. Mice were infected with *S*. *suis* epidemic strain SC-19, which causes STSLS in humans, mice, and pigs, and then treated with the caspase-1 inhibitor (casp1 inh) Ac-YVAD-CHO or PBS as a control at 1 h post-infection. Infection of mice with the strain P1/7, which induces only sporadic cases of meningitis and sepsis in pigs, was used as a control. (A) Cytokine levels in peritoneal lavage fluids at 6 h post-infection were determined using ELISA kits (two-tailed, unpaired *t*-tests, n = 5). (B) The bacterial load in the blood was determined to evaluate the effect of caspase-1 (casp1) signaling on *S*. *suis* clearance (two-tailed, unpaired *t*-tests, n = 5). (C) Blood levels of AST, ALT, LDH, and CK at 6 h post-infection (two-tailed, unpaired *t*-tests, n = 5) (D) H&E staining of infected tissue sections from mice at 6 h post-infection with *S*. *suis* epidemic strain SC-19 with or without casp1 inh treatment. Congestion in the lung and spleen is indicated by a “red arrow”, infiltration of inflammatory cells in the lung is indicated by a “hollow arrow”, vacuolated degeneration in the liver is indicated by a “black arrow”, and necrosis in the liver is indicated by a “yellow arrow”. (E) Survival of mice infected with *S*. *suis* epidemic strain SC-19 with or without casp1 inh treatment (log-rank test, n = 10). (F) Clinical signs of mice infected with *S*. *suis* epidemic strain SC-19 with or without casp1 inh treatment were monitored and scored (two-way RM ANOVA, n = 10). Error bars represented the mean ± standard deviations.

STSLS is characterized by high bacterial burden, an inflammatory cytokine storm, multi-organ dysfunction, and ultimately acute host death [6–8]. In a murine model, *S*. *suis* epidemic strain SC-19 infection induced an acute and extremely high inflammatory cytokine response, including increased IL-1β, IL-18, TNF-α, IL-17A, and IFN-γ levels **(Fig 1A)**, high bacterial burden **(Fig 1B)**, and high CK (creatine kinase), ALT (alanine aminotransferase), AST (aspartate aminotransaminase), and LDH (lactate dehydrogenase) levels in the blood **(Fig 1C),** resulting in evident injury in multiple organs, such as severe congestion and dense infiltration of inflammatory cells in the lung, severe congestion in the spleen, and severe vacuolated degeneration and necrosis in the liver **(Fig 1D)**. In addition, all infected mice presented with severe clinical signs and died within two days (n = 10) **(Fig 1E and 1F)**. Moreover, the level of inflammatory response and organ damage caused by SC-19 is much higher than classical virulent P1/7 strain, which could also cause high mortality [10]. Thus, murine infection with SC-19 mimicked the STSLS observed in humans.

To evaluate the effect of the inflammasome on STSLS, an inhibitor (inh) of casp1, Ac-YVAD-CHO, was intraperitoneally injected into the infected mice 1 h after infection. Ac-YVAD-CHO treatment significantly reduced the IL-1β and IL-18 levels **(Fig 1A)**, indicating that the secretion of IL-1β and IL-18 depended mainly on casp1 activity. In contrast, TNF-α production was not significantly inhibited by the treatment, which suggested that inhibition of the inflammasome with Ac-YVAD-CHO could not significantly inhibit the casp1-unrelated pro-inflammatory cytokine response **(Fig 1A)**. IL-17A and IFN-γ induction was also inhibited by Ac-YVAD-CHO **(Fig 1A)** since these cytokines are reported as downstream effectors of the inflammasome [23–25].

Because the bacterial burden in the blood did not significantly decrease at the given time point **(Fig 1B)**, the decreased inflammatory response was not due to a decreased bacterial load, the trigger for activation of this inflammatory signaling pathway. Furthermore, inhibition of casp1 activity also reduced the levels of CK and AST in the blood **(Fig 1C)**, alleviated inflammation and injury in multiple organs **(Fig 1D),** reduced clinical signs and promoted survival **(Fig 1E and 1F)**. Ac-YVAD-CHO was not an exclusive inhibitor for casp1, and it also exhibited some activity against caspase-4/5 [26], which directly recognized intracellular LPS for non-canonical inflammasome activation [27–29]. Therefore, these data indicate a potential critical role of casp1-based inflammasome activation in STSLS.

### Activation of NLRP3 inflammasome in response to *S*. *suis* infection

**Fig 2:**
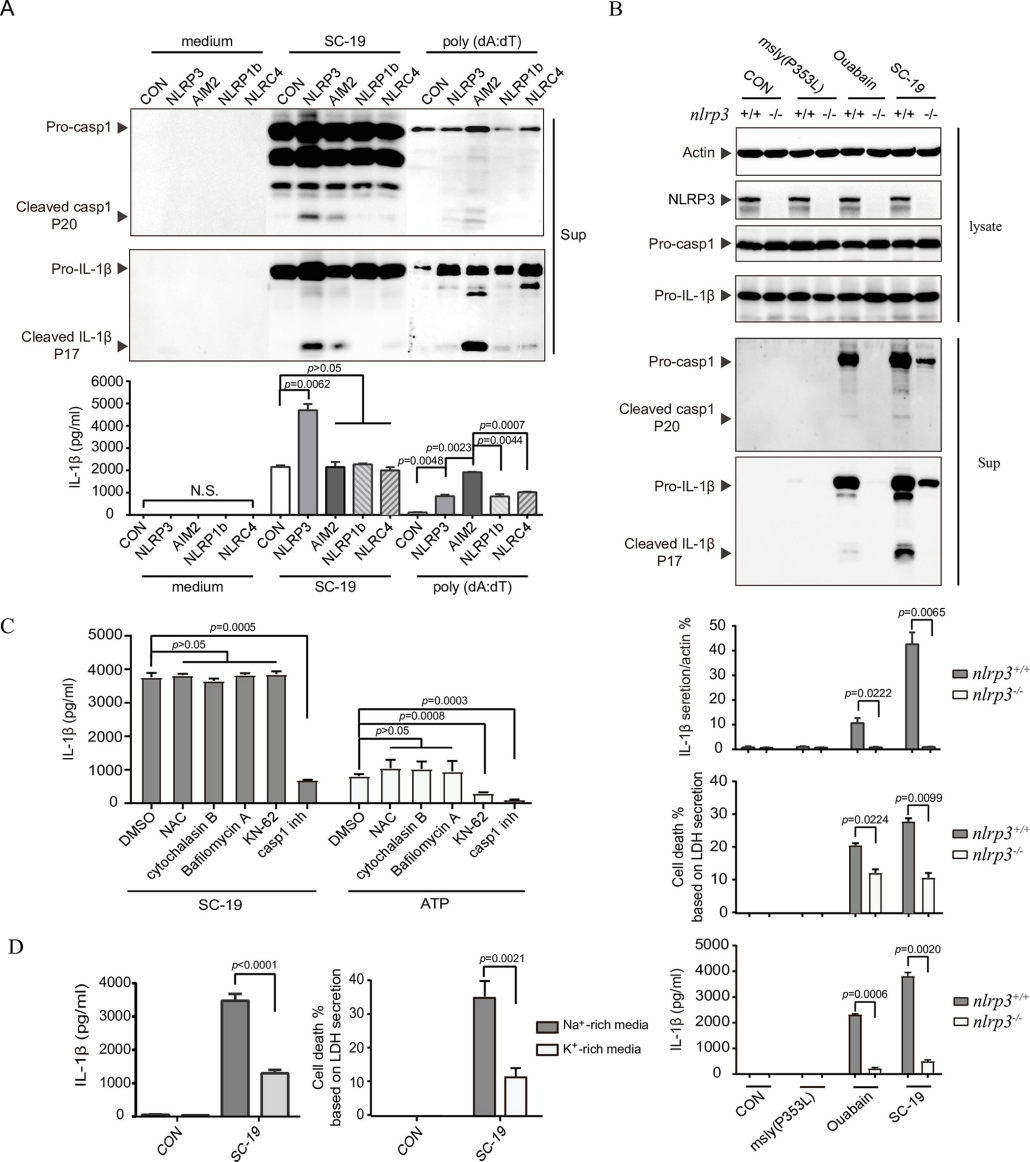
NLRP3 was mainly responsible for inflammasome activation in response to *S*. *suis* infection. (A) 293T cells were transfected with plasmids expressing Myc-tagged ASC, Flag-tagged pro-caspase-1, and Flag-tagged pro-IL-1β and a plasmid co-expressing GFP with NLRP3, NLRP1, NLRC4, or AIM2, followed by infection with *S*. *suis* strain SC-19 or stimulation with poly (dA:dT). Then, the cell supernatants were collected for western blotting with antibodies against casp1 and IL-1β and for the determination of IL-1β with a commercial ELISA kit (two-tailed, unpaired *t*-tests, n = 5). (B) The THP-1 *nlrp3* knockout cell line (THP-1-*nlrp3*^-/-^) and its control cell line (THP-1-*nlrp3*^+/+^) were primed with LPS, followed by infection with *S*. *suis* strains or by stimulation with ouabain. The cellular proteins were subjected to western blot analysis for the expression of actin, NLRP3, casp1 and IL-1β, and the supernatants of cell cultures were collected for detection of casp1 and IL-1β via western blot assay, and the densitometric analysis of mature IL-1β secretion was calculated based on the western blot signal from mature IL-1β in the supernatant / signal from cellular actin. In addition, the IL-1β and LDH concentrations in the supernatants were also determined (two-tailed, unpaired *t*-tests, n = 5). (C) THP-1 cells were primed with LPS, followed by infection with an *S*. *suis* strain or treatment with ATP in the presence of the specific P2X7 antagonist KN-62, the ROS scavenger N-acetyl-L-cysteine (NAC), the phagocytosis inhibitor cytochalasin B, the lysosomal inhibitor bafilomycin A, or the caspase-1 inhibitor (casp1 inh) Ac-YVAD-CHO. IL-1β in the cell culture supernatants with different treatments was detected using a commercial ELISA kit to reflect inflammasome activation (two-tailed, unpaired *t*-tests, n = 5). (D) THP-1 cells were primed with LPS for 4 h and then inoculated in K^+^-rich media or Na^+^-rich media, followed by infection with *S*. *suis*. IL-1β in the supernatants of cell cultures with different treatments was detected using a commercial ELISA kit to reflect inflammasome activation (two-tailed, unpaired *t*-tests, n = 5). Error bars represented the mean ± standard deviations.

To understand the mechanism underlying STSLS development and to identify the type of inflammasome that is activated in response to *S*. *suis* infection, we constructed four types of inflammasome complexes in the 293T cell line **(S1 Fig)**. *S*. *suis* could clearly induce cleavage of pro-casp1 and pro-IL-1β and secretion of IL-1β in 293T cells expressing the NLRP3 inflammasome complex but not in cells expressing the other three (NLRP1, NLRC4, or AIM2) inflammasome complexes **(Fig 2A)**. In contrast, poly(dA:dT) mainly activated the AIM2 inflammasome, as described previously [30] **(Fig 2A)**. These results indicated that NLRP3 was required for inflammasome activation in response to *S*. *suis* epidemic strain SC-19 infection.

To further confirm whether NLRP3 was indispensable for inflammasome activation induced by *S*. *suis*, an *nlrp3*-deficient human acute monocytic leukemia THP-1 cell line (THP-1-*nlrp3*^-/-^) and a control cell line (THP-1-*nlrp3*^+/+^) were constructed using clustered regularly interspaced short palindromic repeats (CRISPR) technology. Similar to cardiac glycosides ouabain, which activates the NLRP3 inflammasome [31], SC-19 infection induced cleavage of pro-casp1 and pro-IL-1β and secretion of IL-1β in THP-1-*nlrp3*^+/+^ cells, but the activation was significantly inhibited in *nlrp3*^-/-^ cells **(Fig 2B)**. This study was also performed using the murine macrophage cell line J774a.1 with *nlrp3* gene knockout (J774a.1-*nlrp3*^-/-^) and the control cell line J774a.1-*nlrp3*^+/+^ **(S2 Fig)**. Thus, NLRP3 was mainly responsible for inflammasome activation induced by *S*. *suis* epidemic strain SC-19 infection.

NLRP3 inflammasome activation can be attributed to several cellular events, including the presence of a P2X7 receptor agonist (extracellular ATP), ROS production, mitochondrial damage, lysosomal damage, formation of large nonspecific pores in the cell membrane, and cytosolic K^+^ efflux [32–34]. Activation of the inflammasome by SC-19 was not inhibited by the single treatment of the P2X7 antagonist KN-62, the ROS scavenger N-acetyl-L-cysteine (NAC), the phagocytosis inhibitor cytochalasin B, or the lysosomal inhibitor bafilomycin A **(Fig 2C),** indicating that inflammasome activation by *S*. *suis* was not dependent on the each single event or was dependent on these complicate events. However, the activation was significantly inhibited in the K^+^-rich media **(Fig 2D)**. Although K^+^ efflux-independent NLRP3 inflammasome activation by small molecules targeting mitochondria had been observed [35], these results indicated that inflammasome activation in response to SC-19 infection was primarily dependent on K^+^ efflux, an essential process for recruitment of NLRP3 to the dispersed trans-Golgi network to cause K^+^-efflux-dependent NLRP3 activation [17].

### The NLRP3 inflammasome was essential for STSLS development

**Fig 3:**
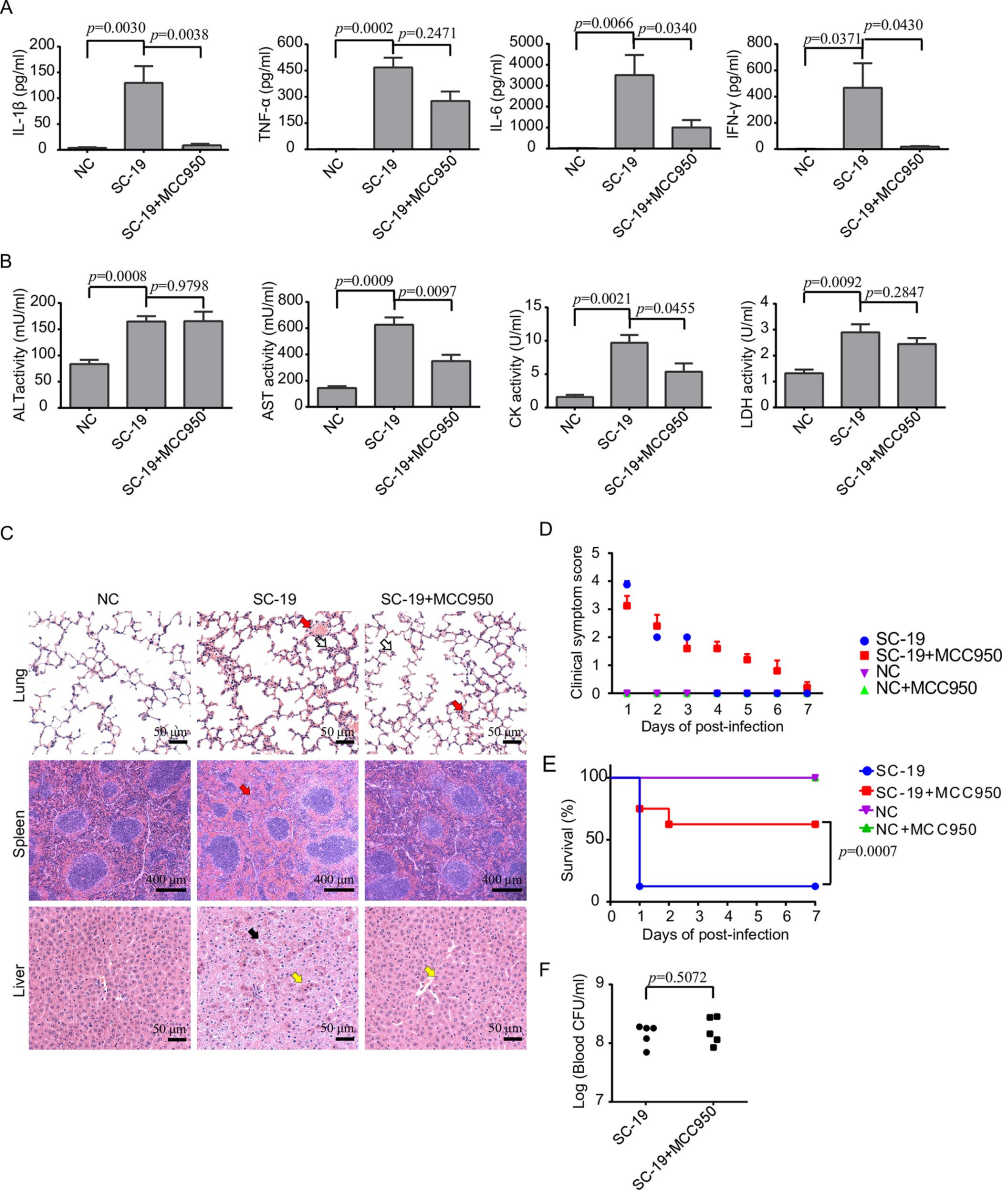
Evaluation of the role of NLRP3 inflammasome activation in STSLS with MCC950. Mice were infected with *S*. *suis* epidemic strain SC-19 and then treated with MCC950 (NLRP3 inhibitor) or control to evaluate the role of NLRP3 in STSLS. (A) Cytokine levels in peritoneal lavage fluids at 6 h post-infection were determined using ELISA kits (two-tailed, unpaired *t*-tests, n = 5). (B) Blood levels of AST, ALT, LDH and CK at 6 h post-infection (two-tailed, unpaired *t*-tests, n = 5). (C) H&E staining of infected tissue sections from mice at 6 h post-infection with *S*. *suis* epidemic strain SC-19 with or without MCC950 treatment. Congestion in the lung and spleen is indicated by a “red arrow”, infiltration of inflammatory cells in the lung is indicated by a “hollow arrow”, vacuolated degeneration in the liver is indicated by a “black arrow”, and necrosis in the liver is indicated by a “yellow arrow”. (D) Clinical symptom scores of mice infected with *S*. *suis* epidemic strain SC-19 and treated with or without the NLRP3 inhibitor MCC950 (two-way RM ANOVA, n = 10). (E) Survival of mice infected with *S*. *suis* epidemic strain SC-19 and treated with or without the NLRP3 inhibitor MCC950 (log-rank test, n = 10). (F) The bacterial load in the blood at 6 h post-infection was determined to evaluate the role of NLRP3 in *S*. *suis* clearance (two-tailed, unpaired *t*-tests, n = 5). Error bars represented the mean ± standard deviations.

Because SC-19 specifically activated the NLRP3 inflammasome *in vitro*, we further investigated the role of NLRP3 in STSLS with a small-molecule inhibitor of the NLRP3 inflammasome, MCC950, which blocks NLRP3-induced ASC oligomerization [36]. MCC950 effectively blocked inflammasome activation by SC-19 *in vitro* **(S3 Fig)**. MCC950 treatment significantly reduced IL-1β level in response to SC-19 infection in mice **(Fig 3A)**. As downstream effects of inflammasome activation, *S*. *suis* infection-induced IL-6 and IFN-γ levels were also significantly decreased by MCC950 treatment **(Fig 3A)**. Therefore, NLRP3 inflammasome activation induced by *S*. *suis* significantly contributed to the inflammatory cytokine storm. MCC950 treatment also reduced the CK and AST levels in the blood **(Fig 3B)**, alleviated injury in multiple organs **(Fig 3C),** decreased clinical signs **(Fig 3D)**, and promoted host survival **(Fig 3E)**, although the bacterial burden in the blood was not significantly changed at the given time point **(Fig 3F).** These indicated that blocking NLRP3 inflammasome could significantly inhibit STSLS caused by SC-19 infection.

**Fig 4:**
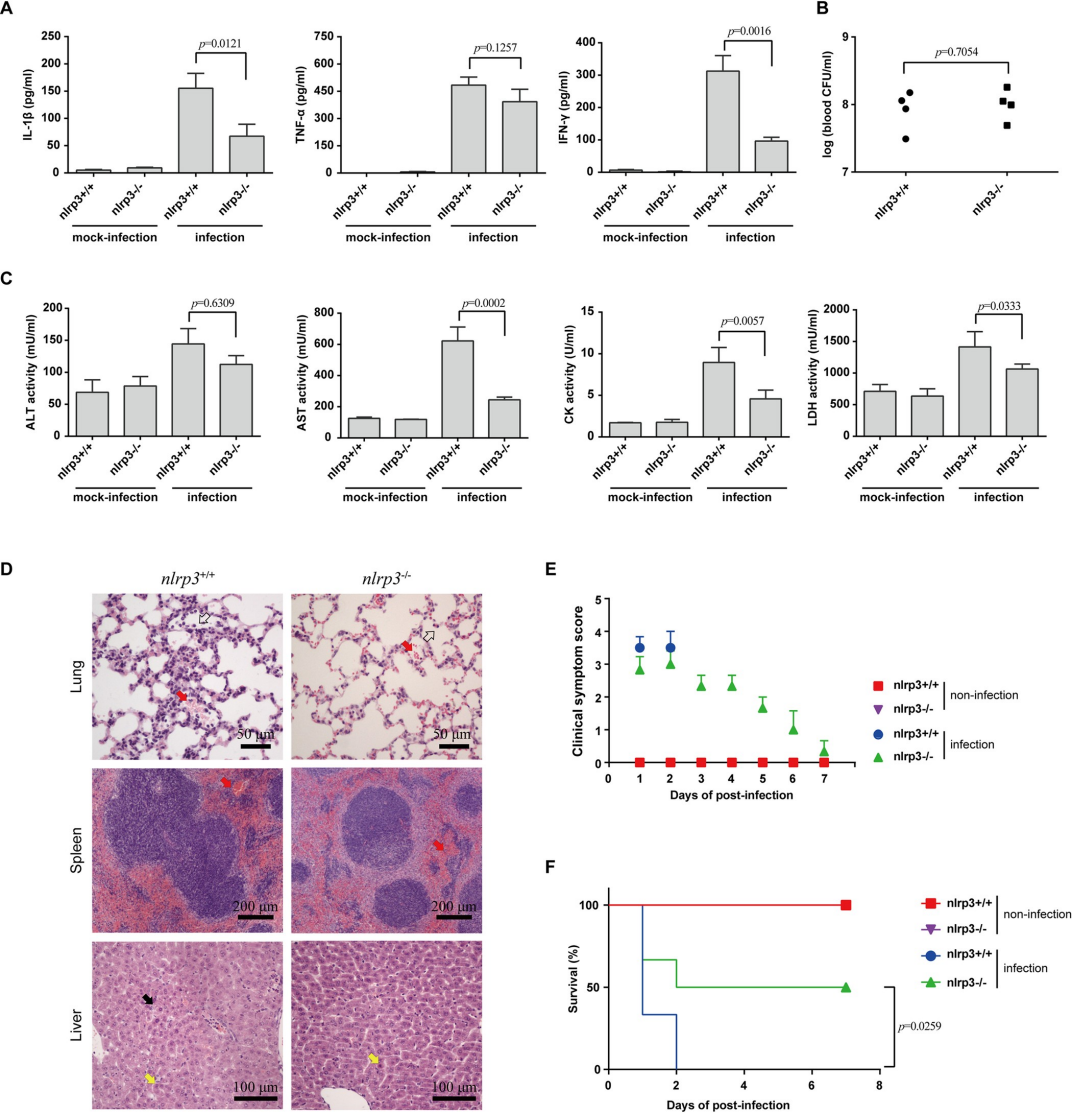
*nlrp3* was required for STSLS caused by *S*. *suis*. The *nlrp3*-deficient mice (*nlrp3*^-/-^) and its wild-type mice (*nlrp3*^+/+^) were infected with *S*. *suis* epidemic strain SC-19. (A) Cytokine levels in peritoneal lavage fluids at 6 h post-infection were determined using ELISA kits (two-tailed, unpaired *t*-tests, n = 4). (B) The bacterial load in the blood at 6 h post-infection was determined (two-tailed, unpaired *t*-tests, n = 4). (C) Blood levels of AST, ALT, LDH and CK at 6 h post-infection (two-tailed, unpaired *t*-tests, n = 4). (D) H&E staining of infected tissue sections from *nlrp3*^-/-^ or *nlrp3*^+/+^ mice at 6 h post-infection with *S*. *suis* epidemic strain SC-19. Congestion in the lung and spleen is indicated by a “red arrow”, infiltration of inflammatory cells in the lung is indicated by a “hollow arrow”, vacuolated degeneration in the liver is indicated by a “black arrow”, and necrosis in the liver is indicated by a “yellow arrow”. (E) Clinical symptom scores of mice infected with *S. suis* epidemic strain SC-19 (two-way RM ANOVA, n = 6). (F) Survival of mice infected with *S. suis* epidemic strain SC-19 (log-rank test, n = 6). Error bars represented the mean ± standard deviations.

To direct investigate the role of NLRP3 in STSLS, the comparison of infection was also performed on *nlrp3*^-/-^ mice and *nlrp3*^+/+^ mice. Similar effects were observed for infection of SC-19 on *nlrp3*^-/-^ mice, it induced significantly decreased levels of IL-1β and IFN-γ comparing to the infection on *nlrp3*^+/+^ mice **(Fig 4A)**, while the bacterial burden in the blood did not significantly decrease at the given time point **(Fig 4B)**. The infection on *nlrp3*^-/-^ also caused significantly decreased levels of CK, AST, and LDH in the blood **(Fig 4C)**, decreased injury in multiple organs **(Fig 4D)**, decreased clinical signs **(Fig 4E)**, and promoted host survival **(Fig 4F)**. These results suggested that the NLRP3 inflammasome activation was essential for STSLS development following epidemic *S*. *suis* strain SC-19 infection.

### SLY of *S*. *suis* activated the inflammasome

**Fig 5:**
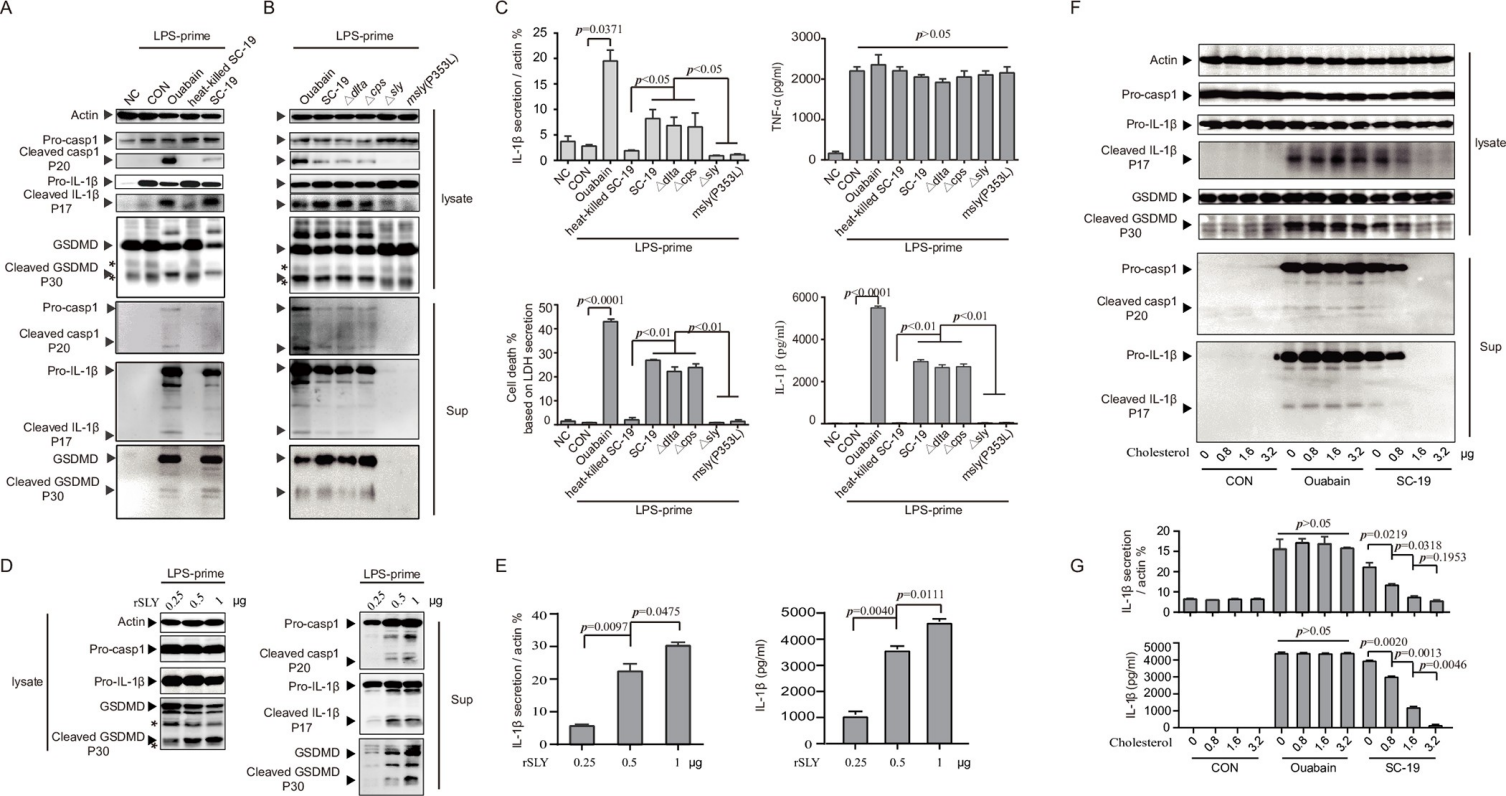
The membrane perforation activity of SLY was mainly responsible for inflammasome activation by *S*. *suis*. THP-1 cells were differentiated into macrophage-like cells by treatment with 50 nM PMA overnight and then primed with LPS for 4 h, followed by infection with *S*. *suis* strains or by stimulation with ouabain or recombinant SLY (rSLY) for 2 h. (A) The THP-1 cells were primed with LPS and then treated with SC-19, heat-killed SC-19 or ouabain. The cellular proteins were subjected to western blot analysis to assess actin, casp1, IL-1β, and GSDMD expression, and the supernatants of the cell cultures were collected for detection of casp1, IL-1β, and GSDMD by western blot assay. Symbols of “black triangle” and “asterisk” indicate the corresponding specific and non-specific protein band. (B) The THP-1 cells were primed with LPS and then treated with ouabain, SC-19 or its isogenic mutants *dlta* (Δ*dlta*), *cpsEF* (Δ*cpsEF*) or *sly* (Δ*sly*) or the mutant strain m*sly* (P353L). The cellular proteins were subjected to western blot analysis to assess actin, casp1, IL-1β, and GSDMD expression, and the supernatants of cell cultures were collected for detection of casp1, IL-1β, and GSDMD by western blot assay. Symbols of “black triangle” and “asterisk” indicate the corresponding specific and non-specific protein band. (C) Densitometric analysis of mature IL-1β secretion was calculated based on the western blot signal from mature IL-1β in the supernatant / signal from cellular actin, and the concentrations of IL-1β, TNF-α, and LDH in the supernatants of THP-1 cells treated with heat-killed or live SC-19, various mutants or ouabain were also detected (two-tailed, unpaired *t*-tests, n = 5). (D) THP-1 cells were primed with LPS and then treated with different concentrations of purified recombinant SLY (rSLY). The cellular proteins were subjected to western blot analysis to assess actin, casp1, IL-1β, and GSDMD expression, and the supernatants of cell cultures were collected for detection of casp1, IL-1β, and GSDMD by western blot assay. Symbols of “black triangle” and “asterisk” indicate the corresponding specific and non-specific protein band. (E) Densitometric analysis of mature IL-1β secretion was calculated based on the western blot signal from mature IL-1β in the supernatant / signal from cellular actin, and the concentrations of IL-1β in the supernatants of THP-1 cells treated with different concentrations of rSLY were detected (two-tailed, unpaired *t*-tests, n = 5). (F) THP-1 cells were primed with LPS and then treated with ouabain or SC-19 in the presence of different concentrations of soluble cholesterol. The cellular proteins were subjected to western blot analysis to assess actin, casp1, IL-1β, and GSDMD expression, and the supernatants of cell cultures were collected for detection of casp1 and IL-1β by western blot assay. (G) Detection of IL-1β in the supernatants of THP-1 cell cultures treated with ouabain or SC-19 in the presence of different concentrations of soluble cholesterol (two-tailed, unpaired *t*-tests, n = 5). “NC” indicates that the cells were not stimulated with LPS, while “CON” indicates that the cells were primed with LPS but not treated with another stimulator. Error bars represented the mean ± standard deviations.

Although we identified the NLRP3 inflammasome as being essential for STSLS development, it was also important to identify the component of *S*. *suis* involved in inflammasome activation. To identify the component of *S*. *suis* involved in inflammasome activation, we found that live, but not heat-inactivated, *S*. *suis* strain SC-19 induced very obvious cleavage of pro-casp1, pro-IL-1β, and GSDMD **(Fig 5A and 5B)**, which resulted in pyroptosis and benefited the secretion of mature IL-1β and IL-18 [19–21]. Furthermore, the secretion of IL-1β was specific because treatment with either live or heat-inactivated *S*. *suis* did not induce significantly more TNF-α at the indicated time point **(Fig 5C)**. Consistent with the results obtained in THP-1 cells, live, but not heat-killed, *S*. *suis* was required for IL-1β secretion, and IL-1β activation was inhibited by the casp1 inh in isolated murine peritoneal macrophages **(S4A Fig)** and bone marrow neutrophils **(S4B Fig)**. Thus, live, but not heat-killed, SC-19 infection activated the inflammasome.

To further identify the component of *S*. *suis* that contributes to inflammasome activation, D-alanylation of lipoteichoic acid (DLTA) [37, 38], the capsular polysaccharides (CPS) structure [39], and SLY [38, 40–42], which are directly involved in the virulence of *S*. *suis*, were selected for evaluation of their roles in inflammasome activation. The isogenic mutants for *dlta* (Δ*dlta*) **(S5 Fig)** or *cpsEF* (Δ*cpsEF*) induced pro-casp1, pro-IL-1β and GSDMD cleavage **(Fig 5B)** and IL-1β secretion **(Fig 5C),** similar to the wild-type (WT) strain. However, the isogenic *sly* mutant (Δ*sly*) completely lost the ability to induce cleavage of pro-casp1, pro-IL-1β and GSDMD **(Fig 5B)** and secretion of IL-1β **(Fig 5C)**, but it did not block TNF-α secretion **(Fig 5C)**. In contrast, the complemental SLY strain could restore the ability for induction of inflammasome **(S6 Fig).** Furthermore, the purified recombinant SLY (rSLY) induced pro-casp1, pro-IL-1β and GSDMD cleavage and IL-1β secretion in a dose-dependent manner **(Fig 5D and 5E and S2 Fig).** These data indicated that SLY of *S*. *suis* activated the inflammasome.

Because SLY is a member of the pore-forming cholesterol-dependent cytolysin family of toxins [43, 44], we further evaluated the role of SLY in inflammasome activation by adding exogenous cholesterol, which can inhibit binding of SLY to host cells [45, 46]. Although cholesterol crystals induced the NLRP3 inflammasome [47], the addition of solubilized cholesterol at the given concentrations inhibited the pro-casp1, pro-IL-1β and GSDMD cleavage **(Fig 5F)** and IL-1β secretion **(Fig 5G)** induced by SC-19 in a dose-dependent manner. In contrast, the addition of solubilized cholesterol at the given concentration did not significantly inhibit the IL-1β secretion induced by the NLRP3 agonist ouabain **(Fig 5F and 5G).** These studies indicated that inflammasome activation in response to *S*. *suis* epidemic strain SC-19 infection required the binding of SLY to host cells.

### The membrane perforation activity of SLY was mainly responsible for inflammasome activation by *S*. *suis*

Structural analysis of *S*. *suis* SLY indicated that P353L would result in a loss of hemolytic activity while retaining the biological activity of erythrocyte aggregation [43], which was further confirmed in a biological experiment using recombinant SLY [45]. To elucidate the mechanism underlying SLY-induced inflammasome activation, we constructed a mutant strain containing the P353L point substitution in SLY [m*sly* (P353L)] to analyze the contribution of the membrane perforation activity of SLY to inflammasome activation **(S7A Fig)**. Compared with WT strain inoculation, m*sly* (P353L) strain inoculation failed to activate the inflammasome **(Figs 2B, 5B and 5C and S2 Fig)**. The inability of m*sly* (P353L) to activate the inflammasome was not due to failed SLY expression, because the amount of SLY in the supernatants of cells treated with m*sly* (P353L) was not less than that in the supernatants of cells treated with the WT strain **(S7C and S7D Fig)**. Therefore, our data strongly suggested that the membrane perforation activity of SLY was very important for inflammasome activation during *S*. *suis* infection.

### The non-hemolytic mutant failed to activate the inflammasome and could not cause STSLS

Previous studies have indicated that SLY may confer bacterial resistance to complement-mediated killing [38, 48] and contribute to enhanced host inflammation [42], which ultimately contributes to *S*. *suis* virulence. The non-hemolytic mutant m*sly* (P353L) retained its resistance to complement-mediated killing, while the Δ*sly* mutant did not **(S7E Fig)**. Therefore, the non-hemolytic mutant m*sly* (P353L) could be used to further confirm the effect of NLRP3 inflammasome activation on STSLS.

**Fig 6:**
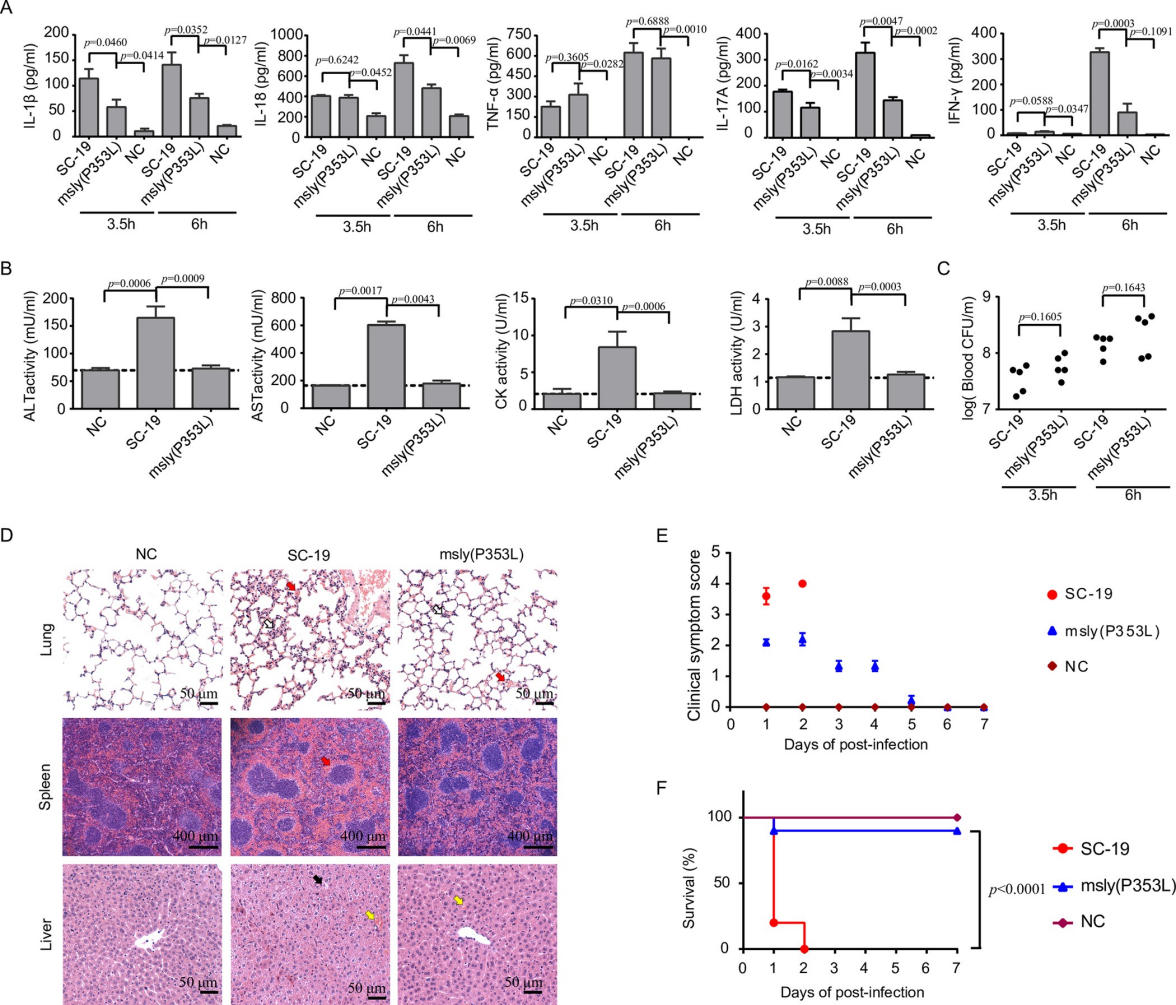
The high membrane perforation activity of *S*. *suis* was required for STSLS. Mice were infected with *S*. *suis* epidemic strain SC-19 or the mutant [m*sly* (P353L)] containing the point substitution P353L, which lacks hemolytic activity. (A) Cytokine levels in peritoneal lavage fluids at 3.5 or 6 h post-infection were detected using ELISA kits (two-tailed, unpaired *t*-tests, n = 5). (B) Blood values of AST, ALT, LDH and CK at 6 h post-infection (two-tailed, unpaired *t*-tests, n = 5). (C) The bacterial load in the blood at 3.5 or 6 h post-infection was detected (two-tailed, unpaired *t*-tests, n = 5). (D) H&E staining of infected tissue sections from mice at 6 h post-infection with *S*. *suis* epidemic strain SC-19 or mutant [m*sly* (P353L)]. Congestion in the lung and spleen is indicated by a “red arrow”, infiltration of inflammatory cells in the lung is indicated by a “hollow arrow”, vacuolated degeneration in the liver is indicated by a “black arrow”, and necrosis in the liver is indicated by a “yellow arrow”. (E) Clinical signs of mice infected with *S*. *suis* were monitored and scored (two-way RM ANOVA, n = 10). (F) Survival of mice infected with *S*. *suis* strains (log-rank test, n = 10). Error bars represented the mean ± standard deviations.

As expected, m*sly* (P353L) did not induce high levels of the inflammasome-regulated pro-inflammatory cytokines IL-1β and IL-18 or the downstream effectors, including IL-17A and IFN-γ, in contrast with the WT strain, but the mutant could still induce comparatively high levels of the inflammasome-unrelated cytokine TNF-α **(Fig 6A)**. Notably, the trend in the induction of these inflammasome-related cytokines by the mutant was similar to the effect on *nlrp3*-deficient mice with SC-19 strain infection **(Fig 4)**. These data suggested that membrane perforation activity was required for inflammasome activation *in vivo* and that inflammasome activation was essential for the development of the inflammatory cytokine storm following SC-19 infection. Interestingly, m*sly* (P353L) infection did not result in high levels of ALT, AST, LDH and CK in the blood **(Fig 6B)**, indicating that the mutant did not cause severe multi-organ injury, an essential aspect of STSLS. Furthermore, the bacterial burden was comparable in mice infected with the SC-19 or its mutant strain at the given time points **(Fig 6C)**, which suggested that the decreased inflammasome activation was not attributable to differential bacterial load.

The SC-19 strain caused severe damage to multiple organs and acute death with severe clinical signs; in contrast, 90% of the mice infected with m*sly* (P353L) survived, and only moderate clinical signs and alleviated organ damage were observed during the study **(Fig 6D–6F)**. These data further confirmed that membrane perforation activity was required for inflammasome activation and full virulence of the epidemic strain SC-19, which can cause STSLS.

In summary, these experiments further supported our hypothesis that the membrane perforation activity of SLY leaded to NLRP3 inflammasome activation that was essential for the induction of STSLS following epidemic *S*. *suis* infection.

## Discussion

Highly virulent *S*. *suis* infection in humans, pigs, and mice induces STSLS, which is characterized by high bacterial burden, a cytokine storm, multi-organ dysfunction, and ultimately acute host death [8, 10, 49]. However, no superantigen responsible for toxic shock syndrome was detected in *S*. *suis* [7], indicating that the mechanism underlying STSLS is different from that of toxic shock syndrome. Although a few studies have indicated that an excessive inflammatory response is responsible for STSLS development [6] and that targeting the pathway may be a potential therapeutic strategy [11, 12], the precise mechanism underlying STSLS remains elusive.

In addition to being a characteristic of acute and fulminating infectious diseases, the “cytokine storm” plays an essential role in the associated high mortality [50, 51]. Therefore, suppression of inflammatory genes is an appealing strategy for preventing death due to severe infections [50]. The “cytokine storm” contributes to STSLS and high mortality [6]; however, the underlying mechanism was unknown. Among these cytokines, IFN-γ plays a broad and important role in severe inflammatory responses and organ injury during shock syndrome [10, 52, 53]. In the present study, NLRP3 inflammasome activation was responsible for high IFN-γ level, multi-organ dysfunction, and mortality in response to epidemic *S*. *suis* infection **(Figs 3–4)**. These findings further demonstrated that NLRP3 inflammasome activation was important for *S*. *suis*-causing cytokine storm.

The pore-forming toxins have been reported to activate the inflammasome through various means [29, 54–56]. For extracellular Gram^-^ bacteria, the toxins could help the bacterial outer membrane vesicles to escape from early endosomes [29], which was important for non-canonical inflammasome activation through caspase-11/4/5 to recognize the intracellular LPS [57, 58]. For extracellular Gram^+^ bacteria, the precise underlying mechanism remains unclear. Inflammasome activation by SC-19 was blocked in K^+^-rich media, which could also inhibit inflammasome activation by *Streptococcus pneumonia* [55]. The present study further indicated that the activation by this toxin could not be inhibited by any one of the inhibitors that block inflammasome activation by extracellular ATP and other stimulators (Fig 2), which indicated that the toxin activated inflammasome through various means. However, the activation by the toxin could be inhibited in the K^+^-rich media **(Fig 2)**, providing a direct explanation for SLY activation of the inflammasome: SLY-induced formation of large pores might cause cytosolic K^+^ efflux-dependent NLRP3 inflammasome activation, which could further result in pro-casp1, pro-IL-1β, and GSDMD cleavage, leading to pyroptosis and facilitating the secretion of mature IL-1β and IL-18 [19–21], which ultimately leads to severe inflammation and STSLS.

In fact, the association of SLY with the virulence of *S*. *suis* has been known for decades [40–42, 59]. Although SLY does not seem to be a critical virulent factor for some strains [40], it is essential for the full virulence of the epidemic strain, which can cause STSLS [60]. SLY was first confirmed to be involved in resistance to complement-mediated killing [38, 48] and to contribute to the virulence of *S*. *suis* [42]. Recently, SLY was demonstrated to be the main stimulus for TNF-α production independently of its membrane perforation ability [61], and it was also involved in the invasive infection caused by *S*. *suis* [46, 62–64]. Here, we demonstrated that SLY was essentially responsible for the high level of inflammasome activation by *S*. *suis* **(Fig 5)** because the isogenic *sly* mutant showed no obvious ability to activate the inflammasome and inflammasome activation was significantly inhibited by soluble cholesterol, the target molecule in the cell membrane for SLY binding [44]. Furthermore, the membrane perforation activity of SLY was indispensable for inflammasome activation **(Fig 5)**. Undoubtedly, all these pathogenic functions of SLY may contribute to the virulence of *S*. *suis* [46, 62, 63, 65, 66]. To further determine the significance of inflammasome activation by SLY for virulence, we constructed the mutant m*sly* (P353L), which expresses SLY with a point mutation that resulted in a defect in hemolytic activity. The strain retained complement-mediated killing ability but lost its membrane perforation activity and the ability to activate the inflammasome **(S7 Fig)**. Interestingly, the mutant maintained its ability to resist bacterial clearance and induced high levels of TNF-α, similar to the WT strain (the epidemic strain), but could not significantly induce high levels of inflammasome-related cytokines, which was similar to the effect of inflammasome inhibitors on *S*. *suis* infection. As a result, the mutant could not cause the cytokine storm and multi-organ failure **(Fig 6)**. Therefore, the present study strongly indicates that the membrane perforation activity of SLY is important for causing high levels of NLRP3 inflammasome activation, which is essential for STSLS development.

However, it is still difficult to explain why the epidemic strain causes STSLS while other *sly*^+^ strains (such as the P1/7 strain) do not. Interestingly, the epidemic strain expressed higher levels of SLY [67], which further activated the inflammasome **(S8 Fig)**. Surprisingly, a novel hemolysis-related gene was identified in the 89K pathogenicity island (89K PI), which could increase SLY expression [68]. Because the 89K PI was specifically present in the genome of the epidemic *S*. *suis* strain [69] and could be transferred in a T4SS-mediated horizontal manner [70], increased SLY expression due to the acquisition of the 89K PI might explain why the epidemic strain suddenly had the ability to cause high level of inflammasome activation and STSLS development. Therefore, it would be worthy to further elicit the mechanism underlying the regulation of SLY by the 89K PI.

**Fig 7:**
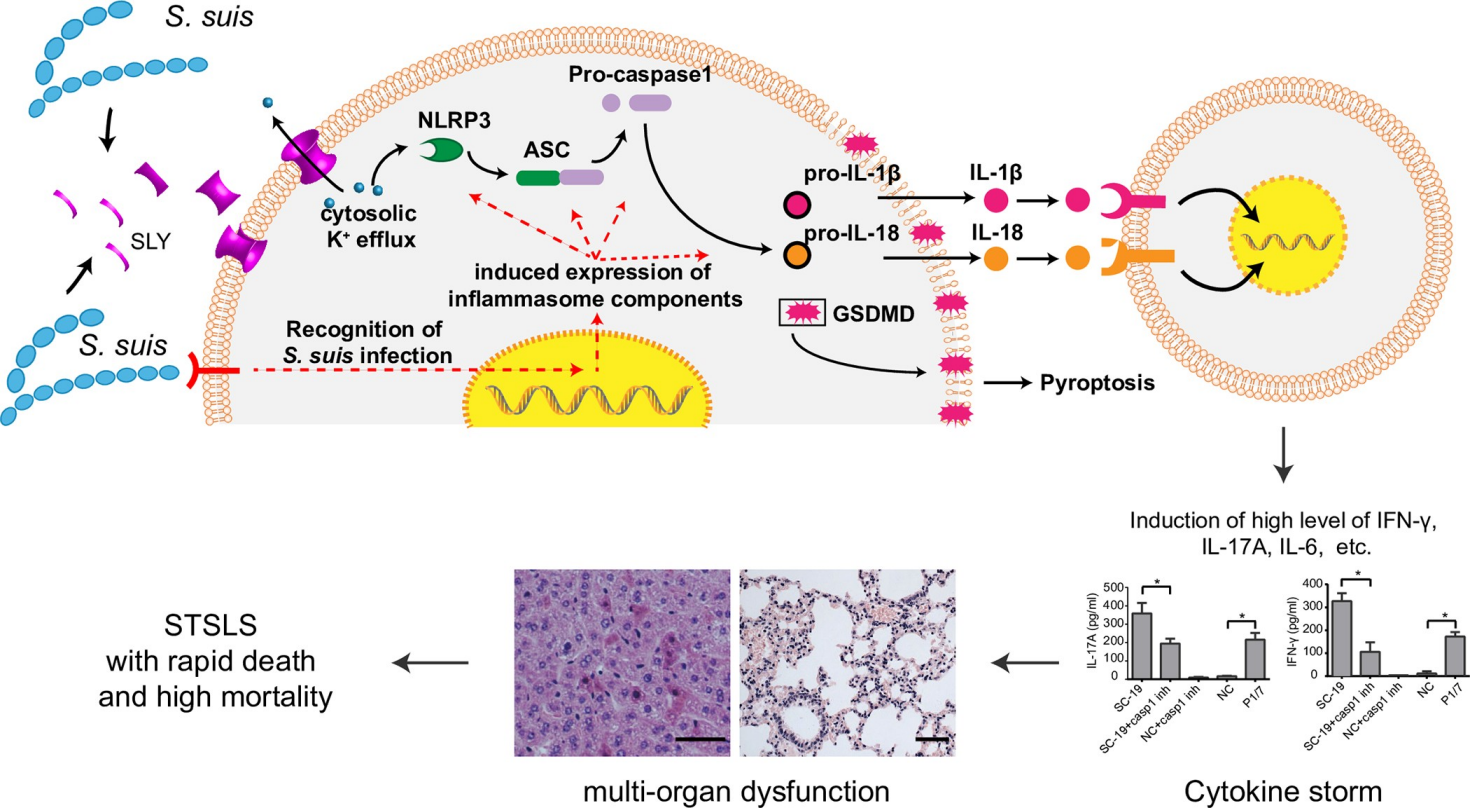
Schematic representation of the mechanism underlying STSLS. The present study indicated an important mechanism by which the epidemic S. *suis* strain causes STSLS. First, infection with *S*. *suis* can activate the transcription of genes involved in the inflammasome through pattern-recognition receptors, such as Toll-like receptor (TLR). Then, the high SLY expression level allows the strain to exert high levels of membrane perforation activity, which can further result in several events, including cytosolic K^+^ efflux, an essential event for NLRP3 inflammasome activation. Subsequently, the high level of inflammasome activation results in GSDMD, pro-IL-1β and pro-IL-18 cleavage, and the GSDMD cleavage leads to pyroptosis and facilitates secretion of mature IL-1β and IL-18, which may further induce the production of downstream cytokines, such as IFN-γ and IL-17A, causing a cytokine storm and multiple organ dysfunction, the main characteristics of STSLS.

In conclusion, we identified an important mechanism by which the epidemic S. *suis* strain causes STSLS **(Fig 7)**. First, *S*. *suis* infection may activate the transcription of genes involved in the inflammasome through pattern-recognition receptors, such as Toll-like receptor (TLR) [9, 61, 71, 72]. Then, acquisition of the 89K PI enables the strain to increase SLY expression, the high membrane perforation activity of which causes several events, including cytosolic K^+^ efflux, an essential event for NLRP3 inflammasome activation. Thus, strong activation of the inflammasome is an important mechanism by which this strain causes the cytokine storm, multi-organ dysfunction, and a high mortality rate, which are hallmarks of STSLS. Therefore, our study provides an explanation for STSLS development and indicates that the NLRP3 inflammasome is an attractive target for the treatment of STSLS.

## Materials and methods

### *S*. *suis* strains

The *S*. *suis* epidemic strain SC-19, which shows high pathogenicity in humans, mice and pigs [11, 73], was used in the present study. The isogenic mutants for *cpsEF* (Δ*cpsEF*) [74], *sly* (Δ*sly*) [75], *dlta* (Δ*dlta*) and a mutant [m*sly*(P353L)] containing a point substitution P353L were originally from strain SC-19 **(S5 and S7 Figs).** The *S*. *suis* strain P1/7, which induces only sporadic cases of meningitis and sepsis in pigs [76], was used as a non-STSLS-causing control. The *sly* gene and its predicted upstream promoter was constructed into a *S*. *suis*-*E*. *coli* shuttle vector pSET2 [77], and then introduced into Δ*sly* strain to obtain the complemented SLY on Δ*sly* strain (Δ*sly*-Csly).

### Ethics statement

The experimental infectious studies were performed in strict accordance with the Guide for the Care and Use of Laboratory Animals Monitoring Committee of Hubei Province, China, and the protocol was approved by the Scientific Ethics Committee of Huazhong Agricultural University (Permit Number: HZAUMO-2015-014). All efforts were made to minimize the suffering of the animals.

### Experimental infections of mice

Five- to six-week-old Balb/c mice with similar body weights were randomly divided into groups of 10 mice and challenged with 0.5 mL of *S*. *suis* strains (8 × 10^8^ CFU/mL) by an intraperitoneal (i.p.) injection to evaluate the pathogenicity of the different *S*. *suis* strains. To evaluate the effect of casp1 and NLRP3 signaling on *S*. *suis* infection, 100 μg of the casp1 inh Ac-YVAD-CHO (Merck Millipore, 400015-1MG, Germany) or PBS as a control; or 37.5 μg of MCC950 (Selleck, S7809, USA), a selective inh of NLRP3, or a PBS control were injected intraperitoneally 1 h post-infection with *S*. *suis*. The experimental infections were also performed on *nlrp3*^-/-^ mice (C57BL/6 background, purchased from the Jackson Laboratory) and *nlrp3*^+/+^ mice (C57BL/6) to direct evaluate the effect of *nlrp3* on STSLS development. All the mice were monitored three times a day for seven days for clinical signs and assigned clinical scores as follows [78]: 0 = normal response to stimuli; 1 = ruffled coat and slow response to stimuli; 2 = respond only to repeated stimuli; 3 = non-responsive or walking in circles; and 4 = dead. Mice exhibiting extreme lethargy or neurological signs (score = 3) were considered moribund and were humanely euthanized.

In addition to the evaluation of mortality, experimental infections were also performed with mice to evaluate the effect of various treatments on the cytokine response, blood biochemistry, and bacterial burden during *S*. *suis* infection. At the indicated time points post-infection with *S*. *suis*, mice in each group were euthanized by carbon dioxide inhalation, and blood was collected via cardiac puncture. Fifty microliters of blood was withdrawn for bacterial load analysis. The remaining blood was used to prepare plasma for analysis of the CK, ALT, AST, and LDH levels with a VITALAB SE Chemistry Analyzer and for analysis of the IL-1β (eBioscience, E09327-1647, USA), TNF-α (eBioscience, E09483-1670, USA), IL-6 (eBioscience, 88-7064-88, USA), IL-17A (eBioscience, 88-7371-88, USA), IL-18 (Sino Biological, SEK50073, China), and IFN-γ (eBioscience, 88-7134-88, USA) levels using commercial ELISA kits. Peritoneal lavage fluid was also collected from each mouse with 2 mL of PBS to analyze the bacterial load and cytokine levels. The lung, kidney, liver and spleen tissues were collected and fixed in 10% neutral buffered formalin and routinely processed in paraffin. Sections with a thickness of 2 to 3 mm were cut for hematoxylin and eosin staining for histopathologic evaluation as previously described [11].

### Bacterial load in the blood and peritoneal lavage

The collected blood samples were serially diluted and then plated on Tryptic Soy Agar plates to evaluate the bacterial load.

### Construction of *nlrp3* gene knockout cell lines

The THP-1 *nlrp3* knockout cell line (THP-1-*nlrp3*^-/-^) was constructed using CRISPR technology [79]. sgRNA (GGATCTTCGCTGCGATCAAC) for human *nlrp3* was designed with an online CRISPR Design Tool (http://tools.genome-engineering.org) and then constructed into a lentiCRISPR v2 vector (Addgene, 52961) to produce the plasmid lentiCRISPR v2-hu*nlrp3*. Then, HEK 293FT cells (ATCC source) were transfected with lentiCRISPR v2-hu*nlrp3*, psPAX2 (Addgene, 12260), and pMD2.G (Addgene, 12259) to produce lentivirus for disruption of the *nlrp3* gene. The lentivirus was then used to transduce THP-1 cells at an MOI = 0.5. After transduction, the THP-1 cells were cultured in the presence of 1μg/mL puromycin (Selleck, S7417, USA) for 5 days. The surviving THP-1 cells were diluted into 96-well plates at a concentration of 1 cell/200 μL and cultured in the presence of 1μg/mL puromycin. The THP-1-*nlrp3*^-/-^ cell line was identified by a western blot assay with NLRP3 antibody (CST, 15101S, USA) and then by DNA sequencing of the *nlrp3* gene. The control cell line (THP-1-*nlrp3*^+/+^) was also constructed according to the same procedure using the original lentiCRISPR v2 plasmid.

The *nlrp3* knockout cell line derived from the murine macrophage cell line J774a.1 (J774a.1-*nlrp3*^-/-^) and its control cell line (J774a.1-*nlrp3*^+/+^) were constructed according to the same procedure used for THP-1 cells. The designed sgRNA targeted the murine *nlrp3* gene and contained the sequence GAAGATTACCCGCCCGAGAA, and the concentration of puromycin for selection of J774a.1-*nlrp3*^-/-^ or J774a.1-*nlrp3*^+/+^ cells was 2.5 μg/mL.

### LDH release assay

Cell supernatants were collected, and LDH release was quantified using a CytoTox 96 Non-Radioactive Cytotoxicity Assay (Promega, USA) according to the manufacturer’s instructions. The percentage of cytotoxicity was calculated based on LDH release in the total cell lysates.

### Measurement of inflammasome activation *in vitro*

THP-1 cells (ATCC source) were differentiated into macrophage-like cells by treatment with 50 nM phorbol myristate acetate (PMA) (Sigma, P8139-1MG) overnight. The differentiated cells (2 × 10^6^ /mL) were primed with LPS (Sigma, L4391) at 0.5 μg/mL for 4 h and then infected with *S*. *suis* strains (2 × 10^7^ /mL) or stimulated with ATP (Sigma, A2383) for 30 min or ouabain (Sigma, O3125) for 2 h in the presence of the following inhibitors: cholesterol (Sigma, C8667-1G), 5 μM cytochalasin B (Sigma, C274), 100 nM KN-62 (Santa Cruz, SC-3560, USA), 2.5 mM NAC (Sigma, 1009005), 50 nM bafilomycin A (InvivoGen, tlrl-baf1, USA), 100 μM casp1 inh, Ac-YVAD-CHO, or the controls containing the corresponding solvents. Then, 100-μL aliquots of the cell culture supernatants were collected to determine human TNF-α (Dakewe Group, DKW12-1720-096, China) and IL-1β (eBioscience, 88-7261-88, USA) secretion levels using commercial available ELISA kits.

The cellular proteins were extracted in Laemmli sample buffer. The proteins in the supernatants were precipitated with 20% trichloroacetic acid on ice for 30 minutes and then washed 3 times with ice-cold acetone. After the last wash, the acetone was removed by vacuum, and the pellets were allowed to air dry for 5 minutes and then dissolved in Laemmli sample buffer. The proteins were subjected to immunoblot analysis with antibodies for the detection of casp1 (Cell Signaling, 3866S, USA), GSDMD (Proteintech, 66387-1-Ig, USA), or IL-1β (Proteintech, 16806-1-AP, USA). Actin was also detected as an internal control using a specific antibody (Proteintech, 66009-1-AP, USA).

The THP-1-*nlrp3*^-/-^ cell line and its control cell line (THP-1-*nlrp3*^+/+^) were also subjected to detection of inflammasome activation according to the procedure described for THP-1 cells.

Inflammasome activation was also performed using the murine macrophage cell line J774a.1 with the *nlrp3* gene knockout (J774a.1-*nlrp3*^-/-^) and its control cell line J774a.1-*nlrp3*^+/+^ via western blotting with antibodies against casp1 (R&D MAB6215, USA) and IL-1β (BIO vision, 5129-30T) and with ELISA kits for TNF-α (eBioscience, E09483-1670, USA) and IL-1β (eBioscience, E09327-1647, USA).

Murine peritoneal macrophages and bone marrow neutrophils were isolated according to a procedure described previously [74]. Detection of inflammasome activation in isolated murine peritoneal macrophages and bone marrow neutrophils was also performed as described for THP-1 cells with ELISA kits for TNF-α (eBioscience, E09483-1670) and IL-1β (eBioscience, E09327-1647).

### Inhibition of IL-1β and LDH release by KCL

THP-1 cells (ATCC source) were differentiated into macrophage-like cells by treatment with 50 nM PMA (Sigma, P8139-1MG, USA) overnight. The differentiated cells (2 × 10^6^ /mL) were primed with LPS (Sigma, L4391) at 0.5 μg/mL for 4 h and then treated with K^+^-rich media containing 45 mM KCL (Sigma, 746436) or Na^+^-rich media containing 45 mM NaCl (Sigma, S5886) for 1 h, followed by treatment with *S*. *suis* strain SC-19 for 2 h. The supernatants of the cells were collected for IL-1β and LDH detection.

### Construction of the NLRP3 inflammasome in 293T cells

293T cells (ATCC source) (1 × 10^6^ /mL) were co-transfected with 0.3 μg, 0.1 μg, and 0.2 μg of expression plasmids encoding human Flag-tagged pro-IL-1β, Flag-tagged pro-casp1, and Myc-tagged ASC, respectively, and with 0.3 μg of plasmid for co-expression of GFP with NLRP3, NLRP1, NLRC4, or AIM2. The expression of these inflammasome components was confirmed by western blotting with a Myc-tag antibody (CST, 2272S, USA) and a FLAG-tag antibody (MBL, M185-3L, USA) and by examination of GFP expression with a fluorescence microscope (Nikon 80I; Tokyo, Japan).

At 24 h post-transfection, cells were infected with *S*. *suis* strain SC-19 for 2 h or transfected with poly(dA:dT) (Invivogen, tlrl-patn, USA) for 12 h. Then, cell supernatants were collected for the western blot assay with antibodies against casp1 (Cell Signaling, 3866S, USA) and IL-1β (Proteintech, 16806-1-AP, USA) and for determination of IL-1β (eBioscience, 88-7261-88, USA).

### Statistical analysis

Unless otherwise specified, the data were analyzed using two-tailed, unpaired *t*-tests. All assays were repeated at least three times, and the data were expressed as the mean ± standard deviations. For the animal infection experiments, comparisons of survival rates and clinical scores were analyzed with a log-rank test or two-way RM ANOVA, respectively, using GraphPad Prism 6. For all tests, a value of *p* < 0.05 was considered the threshold for significance.

## Supporting information

S1 Fig Construction of the NLRP3 inflammasome in the 293T cell line. 293T cells were transfected with plasmids expressing Myc-tagged ASC, Flag-tagged pro-caspase-1, and Flag-tagged pro-IL-1β and a plasmid co-expressing GFP with NLRP3, NLRP1, NLRC4, or AIM2. The expression of these inflammasome components was confirmed by western blot assay with Myc-tag antibody or FLAG-tag antibody or by examination of GFP expression with a fluorescence microscope.
(TIF)

S2 Fig NLRP3 was required for inflammasome activation in response to *S. suis* infection in the murine macrophage cell line J774a.1. (A) Construction of an *nlrp3* knockout murine macrophage cell line J774a.1 (J774a.1-*nlrp3*^-/-^) or control cell line J774a.1-*nlrp3*^+/+^ using CRISPR technology. The expression of NLRP3 in J774a.1-*nlrp3*^-/-^ and J774a.1-*nlrp3*^+/+^ cells was detected, and actin expression was also detected as a control.
(B) DNA sequencing of the *nlrp3* gene in J774a.1-*nlrp3*^-/-^ and J774a.1-*nlrp3*^+/+^ cells. The sgRNA sequence and PAM sequence are shown in blue and red, respectively.
(C) J774a.1-*nlrp3*^-/-^ and J774a.1-*nlrp3*^+/+^ cells were primed with LPS, followed by infection with *S*. *suis* strains or by stimulation with ATP, poly (dA:dT), or recombinant SLY (rSLY). The cellular proteins were subjected to western blot analysis of actin, NLRP3, casp1 and IL-1β expression, and the supernatants of cell cultures were collected for detection of casp1 and IL-1β.
(D) Densitometric analysis of mature IL-1β secretion was calculated based on the western blot signal from mature IL-1β in the supernatant / signal from cellular actin, and the concentrations of IL-1β and TNF-α in the supernatants of J774a.1-*nlrp3*^-/-^ and J774a.1-*nlrp3*^+/+^ cells treated with *S*. *suis* strains, ATP, poly (dA:dT) or rSLY were also detected with commercial ELISA kits (two-tailed, unpaired *t*-tests, n = 5).
“NC” indicates that the cells were not stimulated by LPS, while “CON” indicates that cells were primed with LPS but not treated with another stimulator. Error bars represented the mean ± standard deviations.
(TIF)

S3 Fig Inflammasome activation in THP-1 cells by *S. suis* was inhibited by MCC950. THP-1 cells treated with MCC950 or PBS as a control were infected with *S*. *suis* epidemic strain SC-19, and then, the secretion of IL-1β (A), TNF-α (B) or LDH (C) was detected to evaluate the effect of the NLRP3 inhibitor MCC950 on inflammasome activation by *S*. *suis* (two-tailed, unpaired *t*-tests, n = 5).
“NC” indicates that the cells were not stimulated by LPS, while “CON” indicates that cells were primed with LPS but not treated with another stimulator. Error bars represented the mean ± standard deviations.
(TIF)

S4 Fig Inflammasome activation by *S. suis* in mouse macrophages and neutrophils. Murine peritoneal macrophages (A) or bone marrow neutrophils (B) were primed with LPS for 4 h and then infected with *S*. *suis* strain SC-19 for 2 h. The concentrations of IL-1β and TNF-α in the supernatants of cell cultures were determined (two-tailed, unpaired *t*-tests, n = 5).
“NC” indicates that the cells were not stimulated with LPS, while “CON” indicates that cells were primed with LPS but not treated with another stimulator. Error bars represented the mean ± standard deviations.
(TIF)

S5 Fig Construction and confirmation of the isogenic mutant for *dlta* (Δ*dlta*). (A) Construction strategy for Δ*dlta*, which was derived from the *S*. *suis* epidemic strain SC-19. The sequence flanking *dlta* was cloned into the temperature-sensitive *S*. *suis*-*E*. *coli* shuttle vector pSET4s, and 1374 bp in the *dlta* gene were deleted from the genome.
(B) PCR confirmation of Δ*dlta* with dlta-F and dlta-R primers. A 2347-bp DNA fragment was amplified from the DNA of the WT strain (lane 2), and a 973-bp DNA fragment was amplified from the Δ*dlta* mutant (lane 1). Lane 3 shows a PCR negative control.
(C) The primer sequences for construction and confirmation of Δ*dlta*.
(TIF)

S6 Fig Complemental SLY strain in Δ*sly* could recover the ability to induction of inflammasome. THP-1 cells were differentiated into macrophage-like cells by treatment with 50 nM PMA overnight and then primed with LPS for 4 h, followed by infection with strain SC-19, Δ*sly*, or SLY complemental strain (Δ*sly-Csly*) for 2 h.
(A) The cellular proteins were subjected to western blot analysis to assess actin, casp1, IL-1β, and GSDMD expression, and the supernatants of the cell cultures were collected for detection of SLY, casp1, IL-1β, and GSDMD by western blot assay. Symbols of “black triangle” and “asterisk” indicate the corresponding specific and non-specific protein band.
(B) Densitometric analysis of mature IL-1β secretion was calculated based on the western blot signal from mature IL-1β in the supernatant / signal from cellular actin, and the concentrations of IL-1β in the supernatants of THP-1 cells treated with *S*. *suis* strains were also detected (two-tailed, unpaired *t*-tests, n = 5).
Error bars represented the mean ± standard deviations.
(TIF)

S7 Fig Construction and analysis of a mutant [m*sly* (P353L)] containing the point substitution P353L. (A) Construction strategy for m*sly* (P353L), which was derived from the *S*. *suis* epidemic strain SC-19. The sequence flanking *sly* (353-461aa) was cloned into the temperature-sensitive *S*. *suis*-*E*. *coli* shuttle vector pSET4s, and the 353–461 aa of *sly* were deleted from the genome. Then, the *sly* 353–461 aa sequence containing the P353L substitution was reintroduced into the genome, and the mutant m*sly* (P353L) was obtained.
(B) The primer sequences for construction of m*sly* (P353L).
(C) Expression of SLY in SC-19, m*sly* (P353L) or Δ*sly* was detected using real-time PCR and western blotting with a monoclonal antibody against SLY (two-tailed, unpaired *t*-tests, n = 5).
(D) Hemolytic activity of SLY from SC-19, m*sly* (P353L), or Δ*sly*. The supernatant of *S*. *suis* was collected, and 1% chicken erythrocyte suspension was incubated with the supernatants for 1 h at 37°C. The supernatants were then transferred for spectrophotometric measurement at 540 nm (two-tailed, unpaired *t*-tests, n = 5).
(E) Percent of bacterial killing after a 90-min incubation with murine anticoagulated blood. A total of 1 X 10^4^ *S*. *suis* bacteria were incubated in 1 mL of murine anticoagulated blood for 90 min at 37°C in a 5% CO_2_ environment. After incubation, the cells were lysed with sterile water. Viable bacterial counts were determined by plating the bacteria onto THA. The percent of bacterial killing = 100%—survival bacteria %.
Error bars represented the mean ± standard deviations.
(TIF)

S8 Fig *S. suis* epidemic strain SC-19 produced more SLY than the virulent strain P1/7 and intermediate virulent strain A7, and SC-19 induced significant inflammasome activation and mature IL-1 secretion. (A) Western blot analysis of SLY expression in different *S*. *suis* strains with a rabbit sera against SLY. Cleavage of pro-IL-1β in the supernatants of THP-1 cells was also detected after treatment with different *S*. *suis* strains.
(B) Densitometric analysis of SLY expression was also calculated based on the western blot signal from SLY in the supernatant / signal from cellular actin.
(C) Densitometric analysis of mature IL-1β secretion was calculated based on the western blot signal from mature IL-1β in the supernatant / signal from cellular actin.
(D) IL-1β in the supernatants of THP-1 cell cultures treated with different *S*. *suis* strains for 3 h was also detected using an ELISA kit (two-tailed, unpaired *t*-tests, n = 5).
Error bars represented the mean ± standard deviations.
(TIF)
