# Supplementary figures and images for: An NLRP3 inflammasome-triggered cytokine storm contributes to Streptococcal toxic shock-like syndrome (STSLS)
Source: PLoS Pathog. 2019 Jun 6;15(6):e1007795. doi: 10.1371/journal.ppat.1007795 (PMC6553798; doi:10.1371/journal.ppat.1007795)

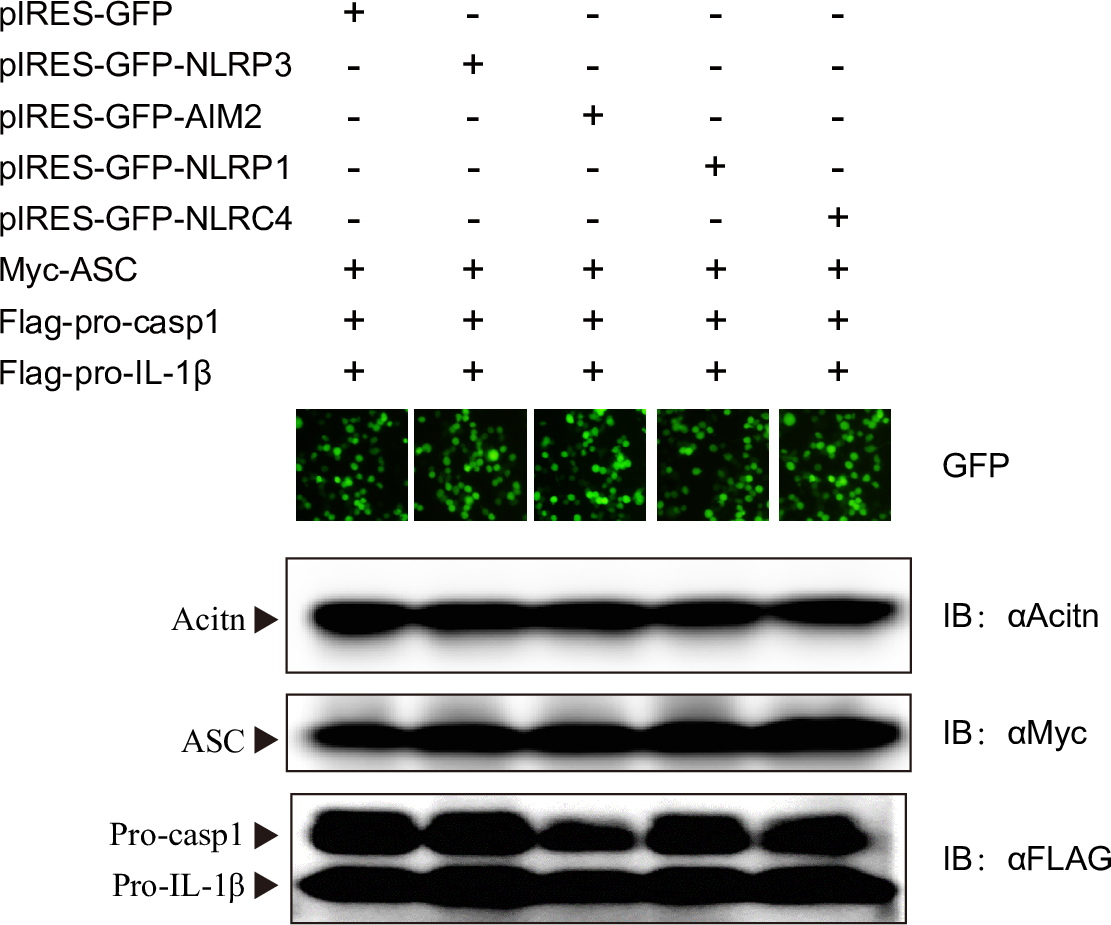

Supplement: S1 Fig — 293T cells were transfected with plasmids expressing Myc-tagged ASC, Flag-tagged pro-caspase-1, and Flag-tagged pro-IL-1β and a plasmid co-expressing GFP with NLRP3, NLRP1, NLRC4, or AIM2. The expression of these inflammasome components was confirmed by western blot assay with Myc-tag antibody or FLAG-tag antibody or by examination of GFP expression with a fluorescence microscope. (TIF) [file ppat.1007795.s001.tif]

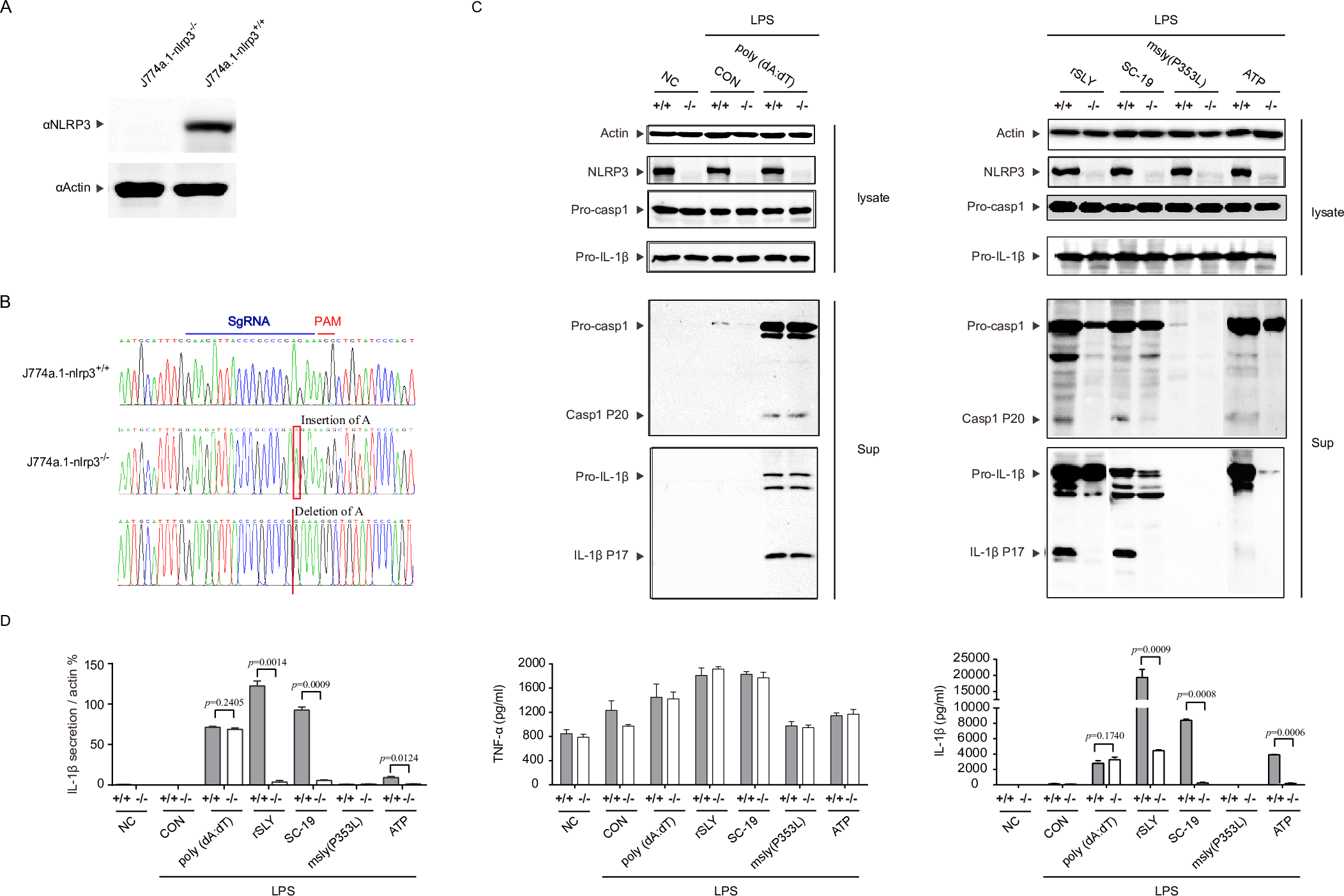

Supplement: S2 Fig — (A) Construction of an nlrp3 knockout murine macrophage cell line J774a.1 (J774a.1-nlrp3-/-) or control cell line J774a.1-nlrp3+/+ using CRISPR technology. The expression of NLRP3 in J774a.1-nlrp3-/- and J774a.1-nlrp3+/+ cells was detected, and actin expression was also detected as a control. (B) DNA sequencing of the nlrp3 gene in J774a.1-nlrp3-/- and J774a.1-nlrp3+/+ cells. The sgRNA sequence and PAM sequence are shown in blue and red, respectively. (C) J774a.1-nlrp3-/- and J774a.1-nlrp3+/+ cells were primed with LPS, followed by infection with S. suis strains or by stimulation with ATP, poly (dA:dT), or recombinant SLY (rSLY). The cellular proteins were subjected to western blot analysis of actin, NLRP3, casp1 and IL-1β expression, and the supernatants of cell cultures were collected for detection of casp1 and IL-1β. (D) Densitometric analysis of mature IL-1β secretion was calculated based on the western blot signal from mature IL-1β in the supernatant / signal from cellular actin, and the concentrations of IL-1β and TNF-α in the supernatants of J774a.1-nlrp3-/- and J774a.1-nlrp3+/+ cells treated with S. suis strains, ATP, poly (dA:dT) or rSLY were also detected with commercial ELISA kits (two-tailed, unpaired t-tests, n = 5). “NC” indicates that the cells were not stimulated by LPS, while “CON” indicates that cells were primed with LPS but not treated with another stimulator. Error bars represented the mean ± standard deviations. (TIF) [file ppat.1007795.s002.tif]

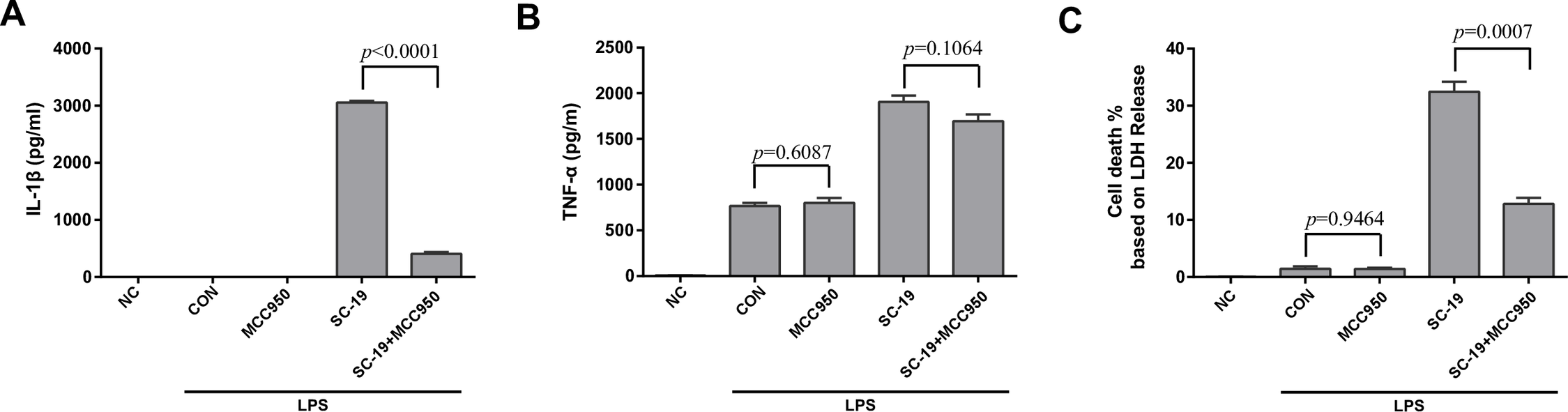

Supplement: S3 Fig — THP-1 cells treated with MCC950 or PBS as a control were infected with S. suis epidemic strain SC-19, and then, the secretion of IL-1β (A), TNF-α (B) or LDH (C) was detected to evaluate the effect of the NLRP3 inhibitor MCC950 on inflammasome activation by S. suis (two-tailed, unpaired t-tests, n = 5). “NC” indicates that the cells were not stimulated by LPS, while “CON” indicates that cells were primed with LPS but not treated with another stimulator. Error bars represented the mean ± standard deviations. (TIF) [file ppat.1007795.s003.tif]

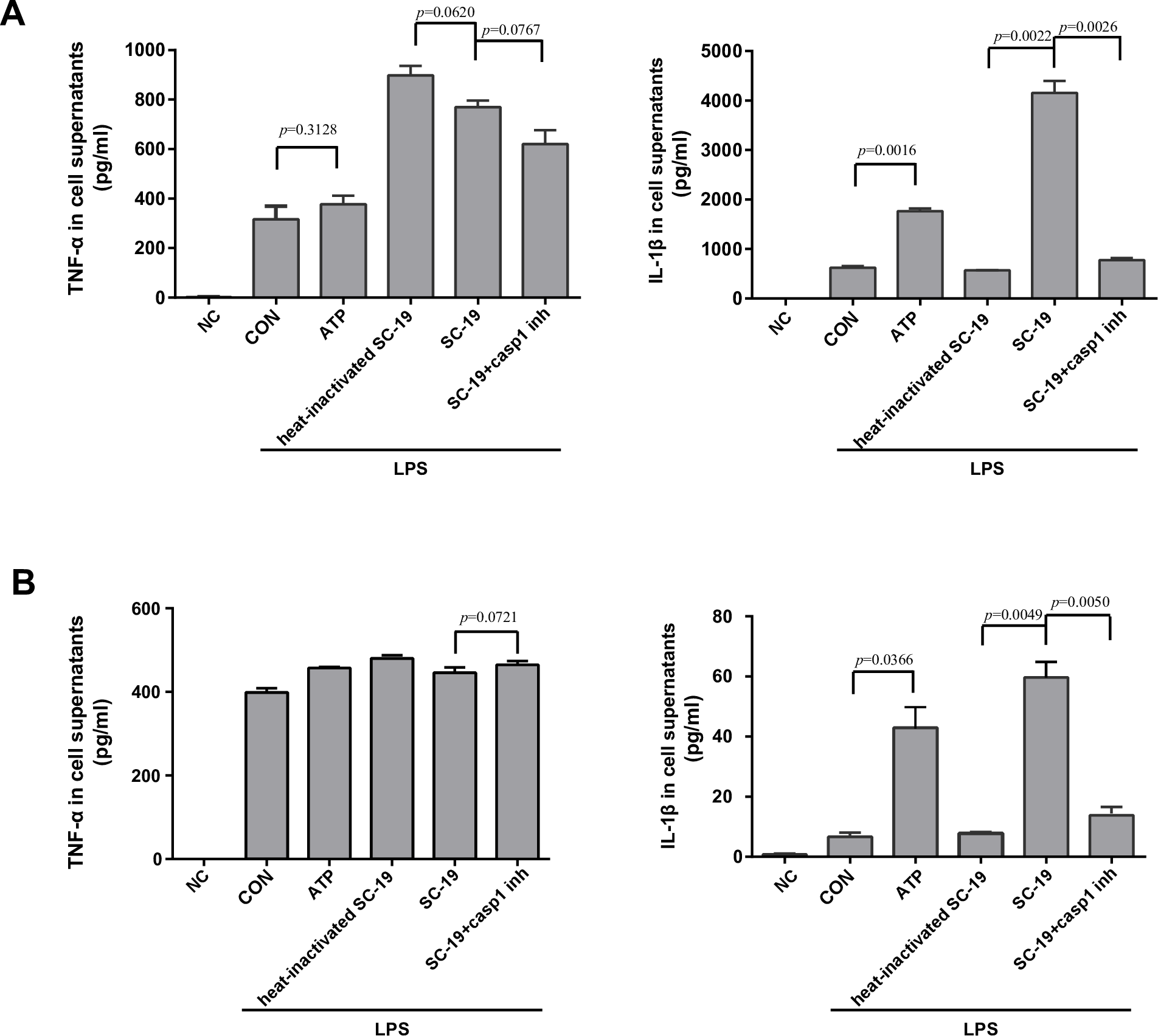

Supplement: S4 Fig — Murine peritoneal macrophages (A) or bone marrow neutrophils (B) were primed with LPS for 4 h and then infected with S. suis strain SC-19 for 2 h. The concentrations of IL-1β and TNF-α in the supernatants of cell cultures were determined (two-tailed, unpaired t-tests, n = 5). “NC” indicates that the cells were not stimulated with LPS, while “CON” indicates that cells were primed with LPS but not treated with another stimulator. Error bars represented the mean ± standard deviations. (TIF) [file ppat.1007795.s004.tif]

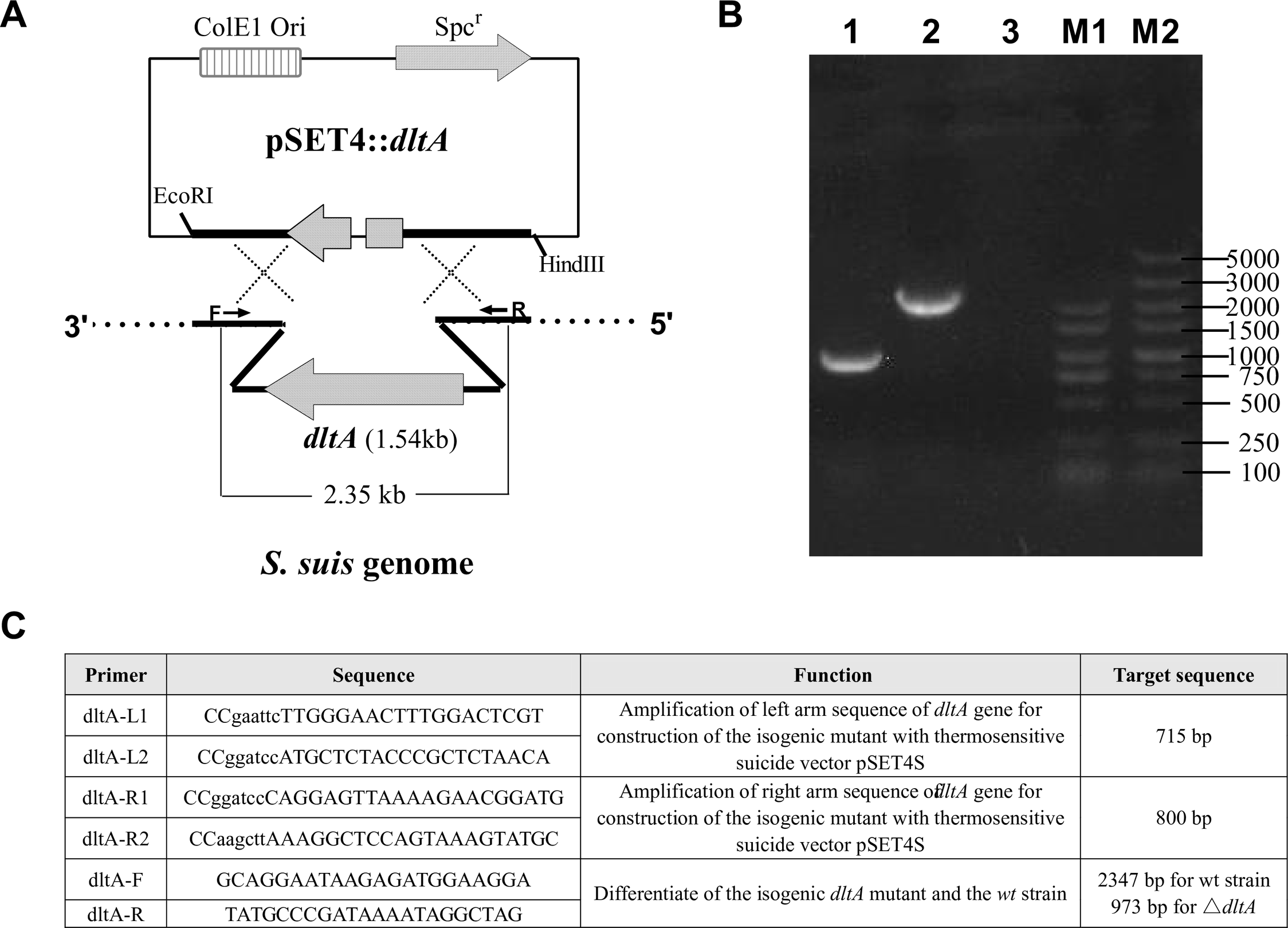

Supplement: S5 Fig — (A) Construction strategy for Δdlta, which was derived from the S. suis epidemic strain SC-19. The sequence flanking dlta was cloned into the temperature-sensitive S. suis-E. coli shuttle vector pSET4s, and 1374 bp in the dlta gene were deleted from the genome. (B) PCR confirmation of Δdlta with dlta-F and dlta-R primers. A 2347-bp DNA fragment was amplified from the DNA of the WT strain (lane 2), and a 973-bp DNA fragment was amplified from the Δdlta mutant (lane 1). Lane 3 shows a PCR negative control. (C) The primer sequences for construction and confirmation of Δdlta. (TIF) [file ppat.1007795.s005.tif]

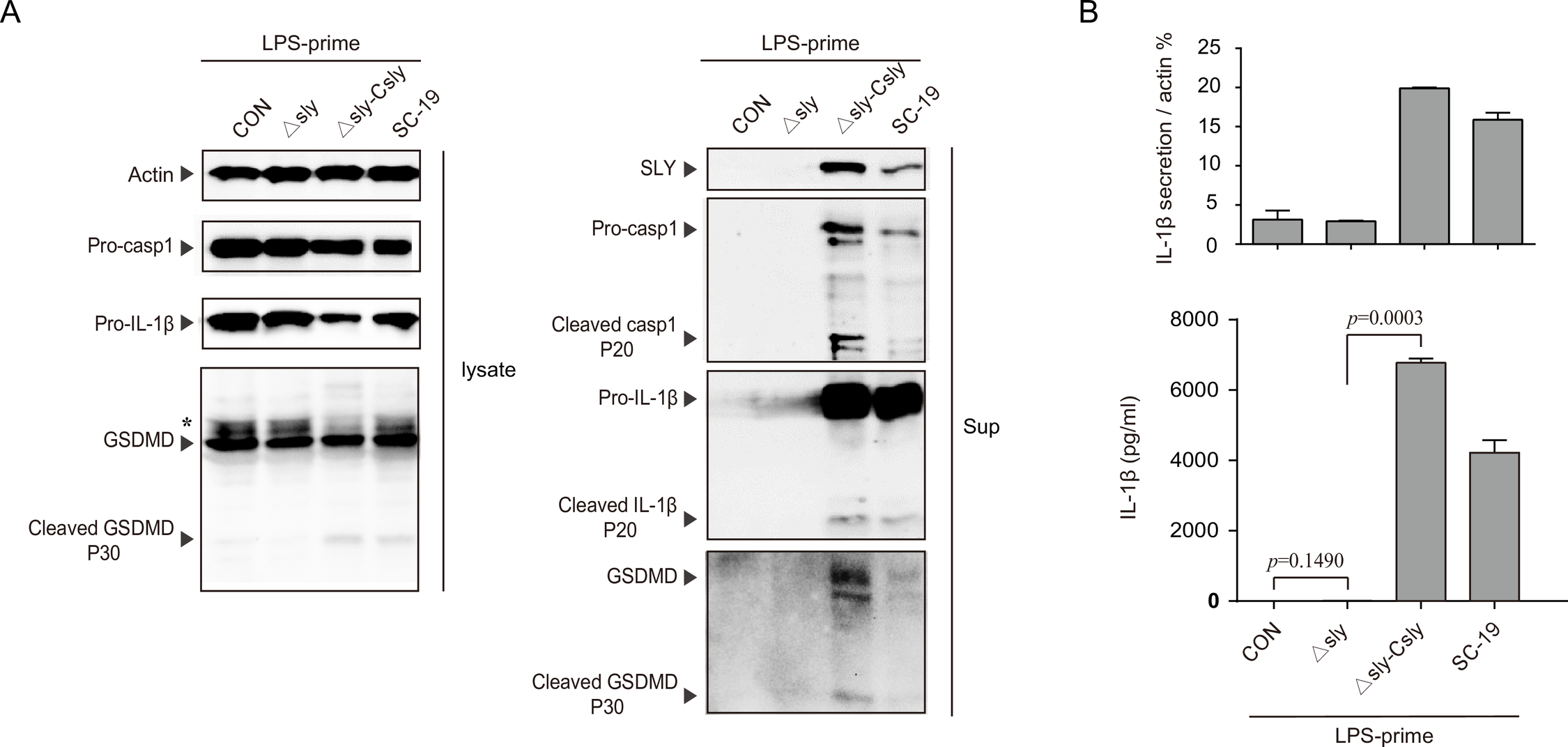

Supplement: S6 Fig — THP-1 cells were differentiated into macrophage-like cells by treatment with 50 nM PMA overnight and then primed with LPS for 4 h, followed by infection with strain SC-19, Δsly, or SLY complemental strain (Δsly-Csly) for 2 h. (A) The cellular proteins were subjected to western blot analysis to assess actin, casp1, IL-1β, and GSDMD expression, and the supernatants of the cell cultures were collected for detection of SLY, casp1, IL-1β, and GSDMD by western blot assay. Symbols of “black triangle” and “asterisk” indicate the corresponding specific and non-specific protein band. (B) Densitometric analysis of mature IL-1β secretion was calculated based on the western blot signal from mature IL-1β in the supernatant / signal from cellular actin, and the concentrations of IL-1β in the supernatants of THP-1 cells treated with S. suis strains were also detected (two-tailed, unpaired t-tests, n = 5). Error bars represented the mean ± standard deviations. (TIF) [file ppat.1007795.s006.tif]

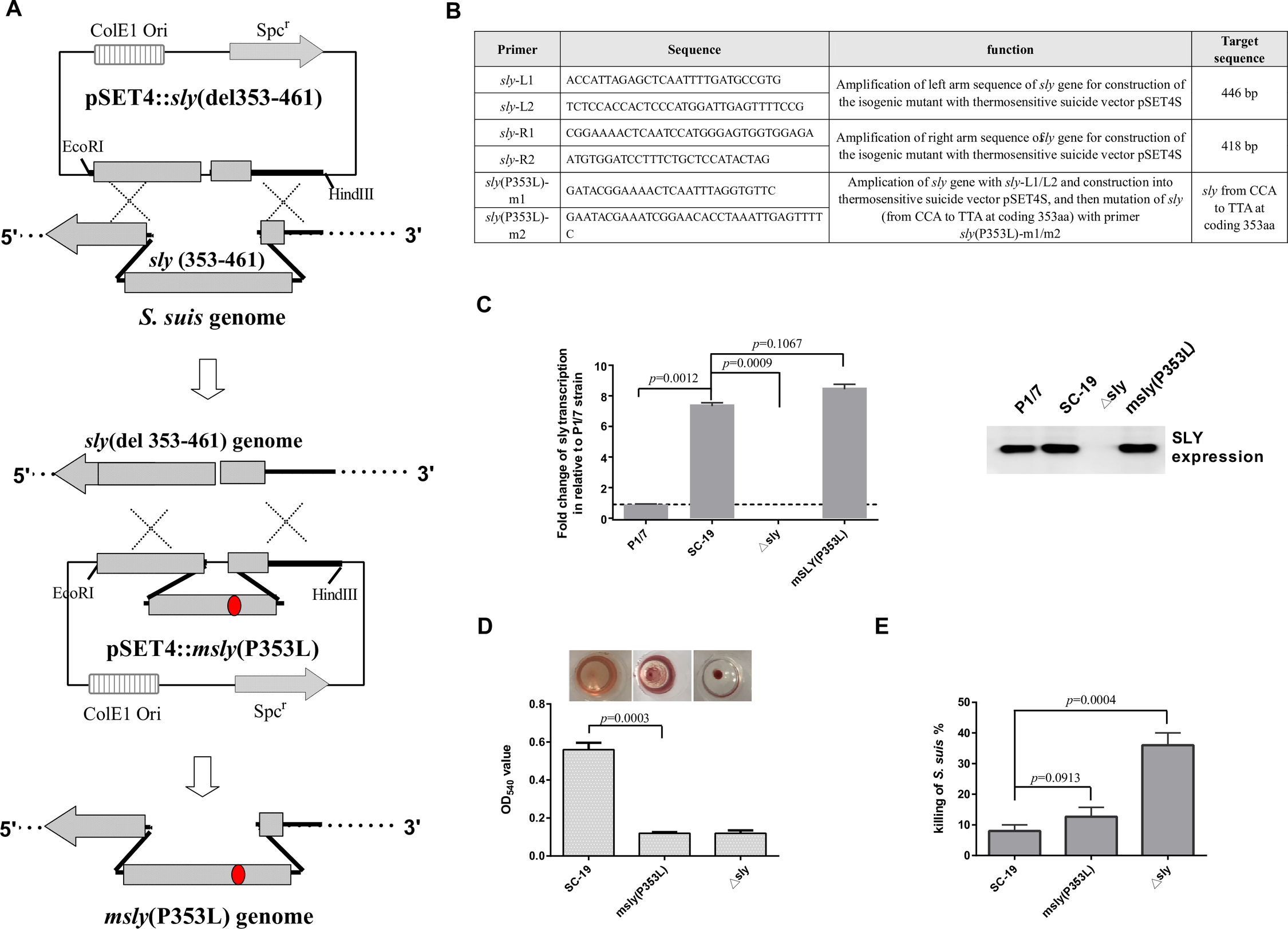

Supplement: S7 Fig — (A) Construction strategy for msly (P353L), which was derived from the S. suis epidemic strain SC-19. The sequence flanking sly (353-461aa) was cloned into the temperature-sensitive S. suis-E. coli shuttle vector pSET4s, and the 353–461 aa of sly were deleted from the genome. Then, the sly 353–461 aa sequence containing the P353L substitution was reintroduced into the genome, and the mutant msly (P353L) was obtained. (B) The primer sequences for construction of msly (P353L). (C) Expression of SLY in SC-19, msly (P353L) or Δsly was detected using real-time PCR and western blotting with a monoclonal antibody against SLY (two-tailed, unpaired t-tests, n = 5). (D) Hemolytic activity of SLY from SC-19, msly (P353L), or Δsly. The supernatant of S. suis was collected, and 1% chicken erythrocyte suspension was incubated with the supernatants for 1 h at 37°C. The supernatants were then transferred for spectrophotometric measurement at 540 nm (two-tailed, unpaired t-tests, n = 5). (E) Percent of bacterial killing after a 90-min incubation with murine anticoagulated blood. A total of 1 X 104 S. suis bacteria were incubated in 1 mL of murine anticoagulated blood for 90 min at 37°C in a 5% CO2 environment. After incubation, the cells were lysed with sterile water. Viable bacterial counts were determined by plating the bacteria onto THA. The percent of bacterial killing = 100%—survival bacteria %. Error bars represented the mean ± standard deviations. (TIF) [file ppat.1007795.s007.tif]

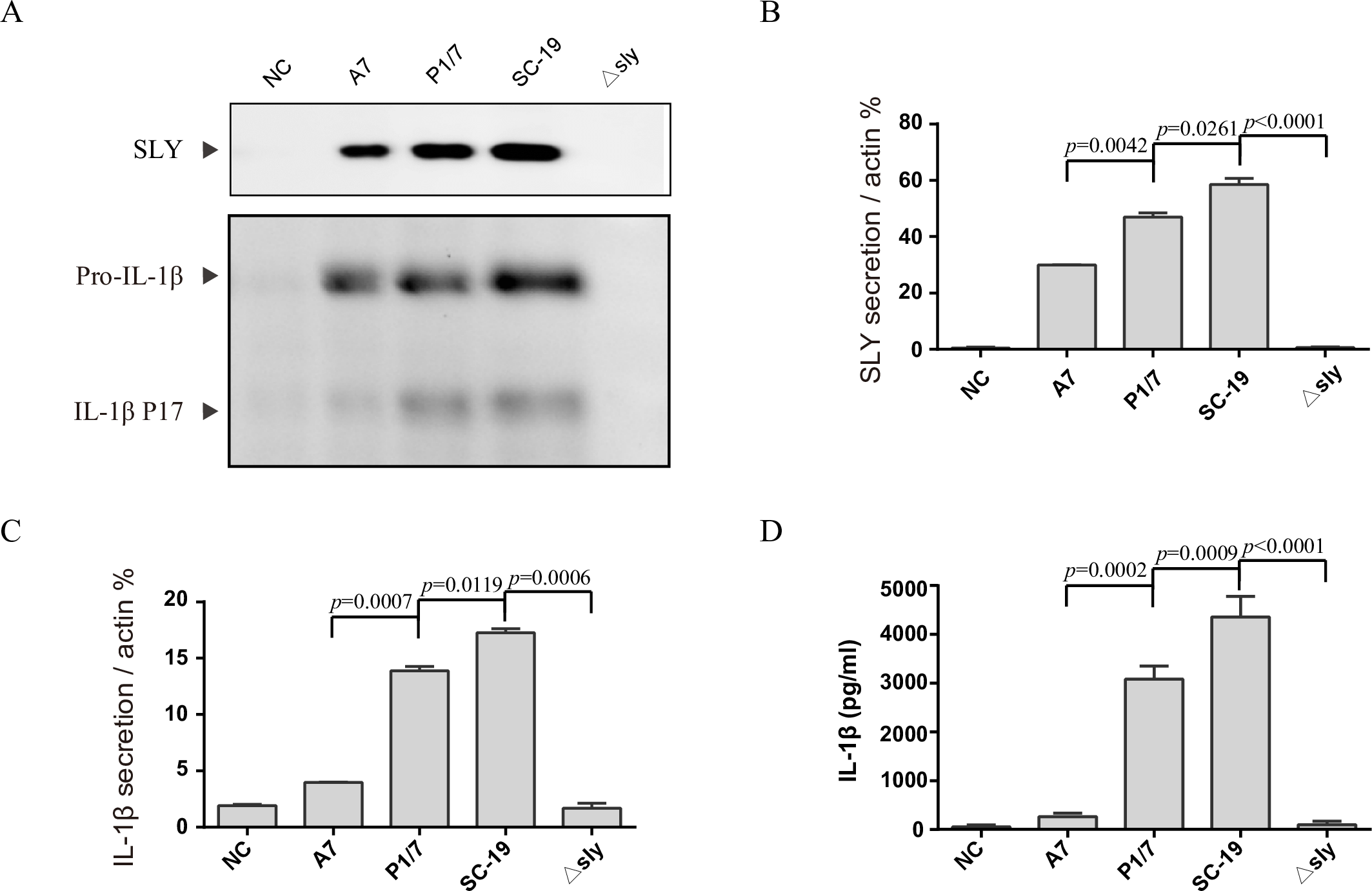

Supplement: S8 Fig — (A) Western blot analysis of SLY expression in different S. suis strains with a rabbit sera against SLY. Cleavage of pro-IL-1β in the supernatants of THP-1 cells was also detected after treatment with different S. suis strains. (B) Densitometric analysis of SLY expression was also calculated based on the western blot signal from SLY in the supernatant / signal from cellular actin. (C) Densitometric analysis of mature IL-1β secretion was calculated based on the western blot signal from mature IL-1β in the supernatant / signal from cellular actin. (D) IL-1β in the supernatants of THP-1 cell cultures treated with different S. suis strains for 3 h was also detected using an ELISA kit (two-tailed, unpaired t-tests, n = 5). Error bars represented the mean ± standard deviations. (TIF) [file ppat.1007795.s008.tif]
